# Effect of Type I Antifreeze Proteins on the Freezing and Melting Processes of Cryoprotective Solutions Studied by Site-Directed Spin Labeling Technique

**DOI:** 10.3390/cryst9070352

**Published:** 2019-07-11

**Authors:** Adiel F. Perez, Kyle R. Taing, Justin C. Quon, Antonia Flores, Yong Ba

**Affiliations:** Department of Chemistry and Biochemistry, California State University, Los Angeles, 5151 State University Drive, Los Angeles, CA 90032, USA

**Keywords:** antifreeze protein, spin labeling, cryo-photo microscopy, electron paramagnetic resonance, cryoprotective agent, ice crystal

## Abstract

Antifreeze proteins (AFPs) protect organisms living in subzero environments from freezing injury, which render them potential applications for cryopreservation of living cells, organs, and tissues. Cryoprotective agents (CPAs), such as glycerol and propylene glycol, have been used as ingredients to treat cellular tissues and organs to prevent ice crystal’s formation at low temperatures. To assess AFP’s function in CPA solutions, we have the applied site-directed spin labeling technique to a Type I AFP. A two-step process to prevent bulk freezing of the CPA solutions was observed by the cryo-photo microscopy, i.e., (1) thermodynamic freezing point depression by the CPAs; and (2) inhibition to the growth of seed ice crystals by the AFP. Electron paramagnetic resonance (EPR) experiments were also carried out from room temperature to 97 K, and vice versa. The EPR results indicate that the spin labeled AFP bound to ice surfaces, and inhibit the growths of ice through the bulk freezing processes in the CPA solutions. The ice-surface bound AFP in the frozen matrices could also prevent the formation of large ice crystals during the melting processes of the solutions. Our study illustrates that AFPs can play an active role in CPA solutions for cryopreservation applications.

## 1. Introduction

Antifreeze proteins (AFPs) and antifreeze glycoproteins (AFGPs) protect organisms living in subzero environments from freezing injury, and even death [1–10]. The mechanism relies on inhibiting the growth of seed ice crystals, and the recrystallization of ice [11]. Type I AFPs have α-helical secondary structures with molecular weights in the range of 3.3–4.5 kDa [4,12–14]. The HPLC6 isoform of type I AFPs [12,13,15–19] has the amino acid sequence: DTASDAAAAAAL TAANAKAAAEL TAANAAAAAAATAR, which contains three 11-residue repeat units beginning with Thr residues [15,20–23]. It was reported that HPLC6 peptides bind to the 12 equivalent bipyramidal planes of ice Ih (hexagonal ice) along the <1 1 0 2> direction [24]. Thus, the binding of the AFP to these surfaces confines the growth of ice crystal to a bipyramidal shape [25–27]. Many experimental results from the studies of mutagenesis and their antifreeze activities [27–33], solid-state NMR [17–19], and site-directed spin labeling technique [34] have demonstrated that the underlined residues in the above sequence bind to the ice surface.

It was also discovered that some northern fishes, such as rainbow smelt, *Osmerus mordax dentex,* produced glycerol in addition to AFPs when the seawater was at subzero temperatures to avoid freezing by increasing the osmolality of their body fluids [35]. The concentration of glycerol was found to be temperature dependent. Because glycerol did not appear to be widely distributed among arctic fishes, it was, thus, suggested that two potential problems might be associated with the smelt antifreeze proteins, including not being entirely effective in stopping ice growth, and being excluded from cells because of its small molecular size, which in turn provided limited freezing protection to cells. Therefore, glycerol might be a solution against freezing damage because it is nontoxic at low concentrations, and could also freely pass through cell membranes to lower the freezing points of water on both sides through colligative effect. It was proposed that antifreeze proteins are the first line of defense against freezing, while glycerol production is reserved as a backup for the time when minimum temperatures were encountered.

AFPs are potentially useful for cryopreservation of living cells, tissues, and organs [36]. Cryopreservation is a method for long-term preservation of living substances at low temperatures without causing fatal freezing damage [37]. Cryoprotective agents (CPAs), such as glycerol, dimethyl sulfoxide, and propylene glycol, have been used as the major ingredients of CPA solutions to treat cellular tissues and organs to prevent the formation of ice crystals, which may cause cell and tissue damages [38]. We believe that the addition of AFPs to CPA solutions could add an additional protective mechanism for cryopreservation applications.

Glycerol was also used as a major ingredient of antifreeze fluids in automotive applications [39] before being replaced by ethylene glycol. The latter has a lower freezing point. Glycerol is non-toxic at low concentration and non-corrosive as well, while can withstand relatively high temperatures. Deicing and anti-icing fluids, used in surface applications to melt ice and prevent ice formation in winter, respectively, are typically composed of ethylene glycol or propylene glycol, along with other additives, such as thickening agents, surfactants, corrosion inhibitors, and colored UV-sensitive dyes. These additives could be toxic to environments. Ethylene glycol is toxic to living organisms, and could remain in the ground even during rainfall because is has high adhesion to soil. Propylene glycol is considerably less toxic, and has lower adhesion to soil. Thus, propylene glycol has high mobility in soil and potentially leaches into groundwater, while being rapidly degraded in all environmental media [40]. Research in the area of antifreeze fluidics is significantly towards the direction for ecological environment. Thus, the use of AFPs as an active ingredient in deicing and anti-icing fluids could enhance their antifreeze property, and meanwhile is eco-friendly.

In this study, site-directed spin labeling (SDSL) technique [34,41–49] was used to study the effect of type I AFPs on the freezing and melting processes of solutions comprised of glycerol, and propylene glycol, respectively, through analyzing the cryo-photo microscopic images of ice crystals, and the EPR (electron paramagnetic resonance) spectra. The molecular information obtained from the EPR spectra and the microscopic information from the observed ice crystals provide complementary information to show how the AFP interacted with ice surfaces and the surrounding solutions during the freezing and melting processes.

## 2. Materials and Methods

### 2.1. Materials

The wild Type I AFPs were purchased from A/F Protein Inc. (Waltham MA, USA) (99%). The cysteine-substituted HPLC6 isoforms of type I AFPs in the 23rd leucine residue (L23C), and the 17th alanine residue (A23C), respectively, were synthesized by Biomatik Corporation (Wilmington, Delaware). The purities of the peptides are > 95%. L23C has the following sequence DTASDAAAAAAL TAANAKAAAEC TAANAAAAAAA TAR, and A17C has the following sequence DTASDAAAAAAL TAANCKAAAEL TAANAAAAAAA TAR, where the underlined residues bind to ice surface [17–19,34,50–53]. The L23C cysteine side chain points to the water phase [34] after binding to the ice surface, while the A17C cysteine replaced the ice binding alanine residue. The MSL (4-maleimido-2, 2,6,6-tetramethyl-1-piperidinyloxy) spin label was purchased from Sigma-Aldrich (CAS Number 15178–63-9). Dialysis tubes with a membrane mesh size of 1 kDa were purchased from Sigma-Aldrich (CAS Number PURD10005–1KT). Phosphate buffered saline tablets (Cas # BP2944–100) were purchased from Fisher Scientific to make the buffer solution at pH 7.4.

### 2.2. Syntheses of the Spin Labeled HPLC6 Peptides

One (1.0) mg of L23C peptides were dissolved in a 0.5 mL pH 7.4 buffer solution held in an Eppendorf vial (2-mL volume), and 1.565 mg of MSL was dissolved in a 0.25 mL 100% ethanol. The two solutions were mixed in a vial. The vial was wrapped with aluminum foil, and the reaction took place under shaking conditions via a vortex mixer for 2–4 h in a 4 °C cold room. Then, the sample was lyophilized. The obtained solid powder was dissolved in 0.5 mL deionized (DI) water and transferred into a dialysis tube. The dialysis was done in DI water at 4 °C. Several changes of water were made at 1 h, 2 h, 4 h, 6 h, and overnight. The dialyses were continued for a longer time (if needed) until no EPR signal could be detected in the DI water. The purified products were lyophilized and stored in a −20 °C freezer.

A MALDI-TOF mass spectrometer (Voyager-DE™ STR Biospectrometry™ Workstation) was used to verify the masses of the spin-labeled L23C and A17C HPLC6 mutants. The matrix was comprised of alpha-cyano-4-hydroxycinnamic acid, 3% TFA, acetonitrile, and DI water. The mass spectra of the original L23C and A17C peptides, and their spin-labeled products have been provided in the supplemental file of our previous publication [34].

### 2.3. EPR Experiments and Line-Shape Simulations

Three solutions were analyzed by the EPR method, including 2.0 mg/mL of the spin-labeled L23C peptide in DI water, that in 5 (volume) % glycerol, and that in 5 (volume) % propylene glycol. Solutions were placed in individual capillaries and fire sealed. The capillaries were immersed in hexane solvent in EPR tubes.

EPR experiments were carried out using a Bruker X-Band CW EMX EPR Spectrometer (Bruker BioSpin Corporation, Billerica, MA, USA). The following EPR parameters were used: Center field—3360.880 G; sweep width—300.00 G; resolution—1024 points; time constant—0.640 ms; sweep time—5.243 s; modulation frequency—100.0 kHz; and modulation amplitude—1.00 G. X-band frequencies from 9.445 to 9.460 GHz were used. Sample temperatures were controlled using an ER 4141VT-UM nitrogen variable-temperature system (77–500 K). EPR temperature readings were corrected using a thermocouple with iced water. Samples were first run at room temperature, then at lower temperatures using heater-regulated cold nitrogen gas generated from a liquid-nitrogen tank. When the assigned temperature was reached (usually in 5–10 min, depending on the cooling conditions), the temperature was maintained for five more minutes. We repeatedly ran the EPR spectra at the same temperatures for a longer time to ensure that the EPR spectra did not change any further after 5 min.

Rotational correlation times of the spin label, along with the component line-shapes, were calculated for each EPR spectrum. Theoretical simulations of the EPR spectra of the spin-labeled peptides were performed using the Multi-Component EPR Fitting program (version 742) and LabVIEW software. This EPR simulation program was developed by Dr. Christian Altenbach (University of California, Los Angeles, CA, USA) [54–56].

### 2.4. Cryo-Photo Microscopic Method

Optical observations of the ice crystals in the AFP solutions were carried out with a set of equipment, including a custom-built Otago nanoliter osmometer, a thermoelectric temperature controlling device with a temperature-controlled cooling stage, an Olympus BX 51 microscope (maximum magnification 800× with a resolution of 1 micron), and a RETIGA 2000R Color Video Camera. The temperature sensor is placed directly on the sample holder; thus, providing a sensitive temperature probe with the accuracy of the temperature reading within ±0.01 °C. The temperature was controlled through the electrical thermostat on the stage, and the cooling process was assisted by heat exchange with 50% isopropyl flowing through tubing at −15 °C by the aid of two cooling pumps cooling at −2 °C and −20 °C, respectively.

## 3. Results and Discussion

### 3.1. The Growths of Ice Crystals in the Spin-Labeled L23C and A17C CPA Solutions

**Figure 1.**
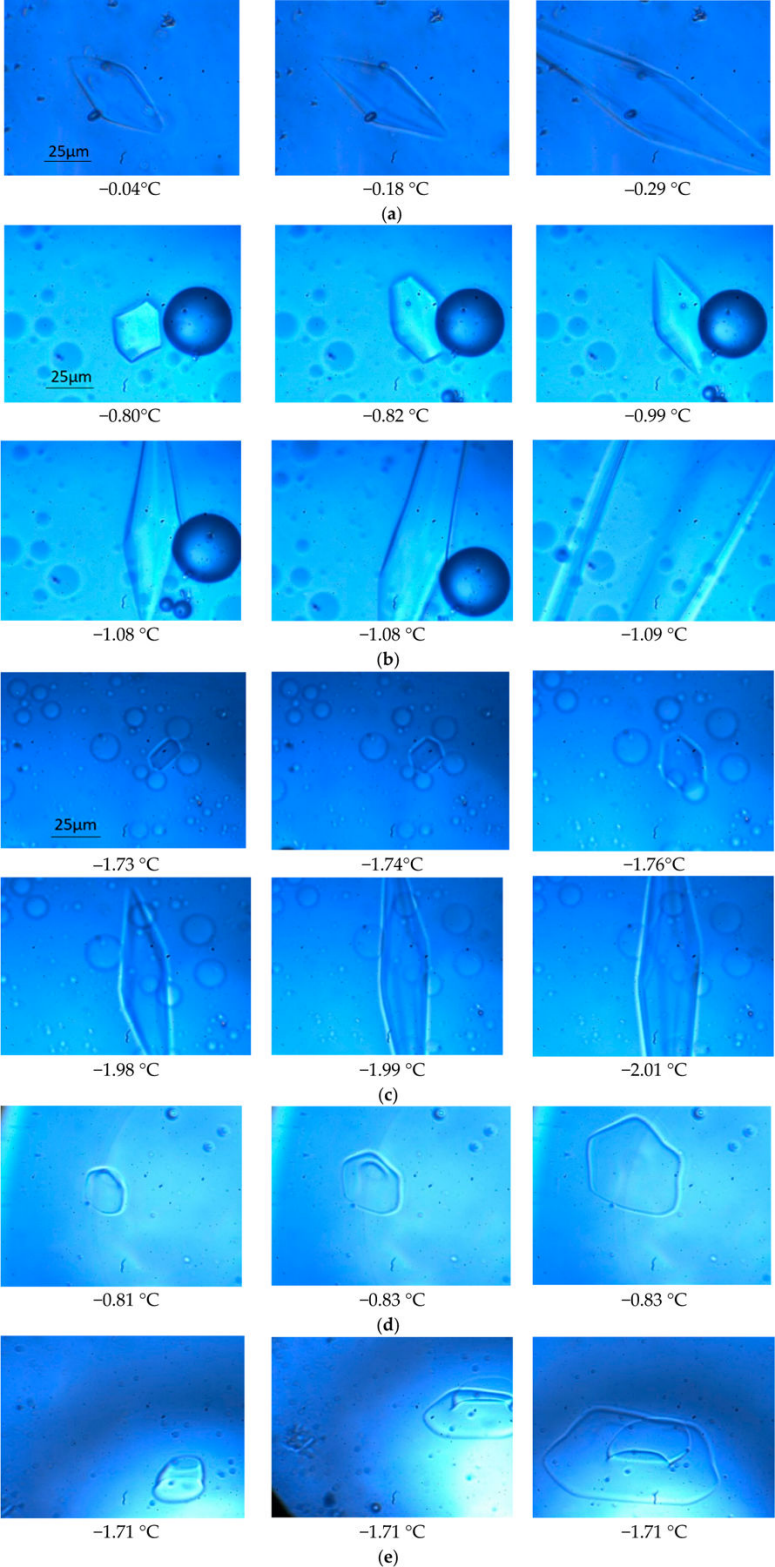
Cryo-photo microscopic images of ice crystals confined by 2.0 mg/mL spin-labeled L23C in (**a**) water; (**b**) 5% glycerol solution, and (**c**) 5% propylene glycol solution, and (**d**) and (**e**) cryo-photo microscopic images of ice crystals grown in the 2.0 mg/mL spin labeled A17C in the 5% glycerol solution, and 5% propylene glycol solution, respectively. (The differences in hue were caused by different filters used when the images were taken, and the bubbles were caused by the dissolved air in the solutions.)

A seed ice crystal in an AFP solution was initiated by rapidly decreasing the temperature of the sample approximately four degrees per second until reaching −10 °C, then continue decreasing until reaching −20 °C with a rate of six degrees per second, referred to as flash-freezing. After flash freezing the whole solution to a low temperature (<−20 °C), the temperature was raised to −10 °C at a rate of four degrees per second, then the temperature was slowly increased to the melting point of the frozen bulk solution. From this point on, the temperature was manually controlled with a rate of ±0.01 °C. The temperature was then varied around the bulk melting point to capture a seed ice crystal. A seed ice crystal had to grow on the basal planes with the decrease in temperature due to the binding of AFP to the other facets, and its shape was finally confined to a bipyramid by the AFP while the rest of the solution stayed in super-cooled condition. Further lowering the temperature to a point allowed the ice crystal to grow abruptly (the so-called ‘burst’ in literature) typically from the tips of the bipyramid and spreading into the whole solution. Figure 1a shows the cryo-photo microscopic images of the growth of an ice crystal in a 2.0 mg/mL spin-labeled L23C water solution (solution a), (b) shows those in a 2.0 mg/mL spin-labeled L23C in a 5% glycerol solution (solution b), and (c) shows those in a 2.0 mg/mL spin-labeled L23C in a 5% propylene glycol solution (solution c). All the ice crystals were confined to bipyramidal shapes when the temperatures were decreased to some points, and the ice crystals eventually burst into the solutions at further lowered temperatures. We define the *antifreeze activity* of an AFP as the difference between the melting point of the single ice crystal and the bursting point of the single ice crystal in an AFP solution. (The antifreeze activity so defined was also called thermal hysteresis in most other publications. Here, we focus on the behavior of a single ice crystal in an AFP solution, while the definition of thermal hysteresis emphasizes the bulk freezing property of an AFP solution.) For example, the melting point of the single ice crystal in the spin labeled AFP water solution was 0.0 °C, and the bursting point of the ice crystal was −0.29 °C. Thus, the antifreeze activity of the spin labeled AFP in water is 0.29 °C in the 2.0 mg/mL AFP solution. The melting points of the other two ice crystals in solution (b) and solution (c) were −0.79 °C and −1.70 °C, respectively, and the corresponding bursting points of these single ice crystals were −1.08 °C and −1.99 °C, resulting in antifreeze activities of 0.29 °C in both of the CPA solutions. To compare with the negative control, spin labeled A17C, we have also studied the behaviors of ice crystals in the A17C CPA solutions. Figure 1d,e show the growths of ice crystals in a 2.0 mg/mL spin-labeled A17C in the 5% glycerol solution, and those in the 5% propylene glycol solution, respectively. As expected, the seed ice crystals grew into sheet-like shapes, showing that the ice crystals grew primarily on the prisms because the spin labeled A17C did not bind to the ice surfaces.

**Figure 2.**
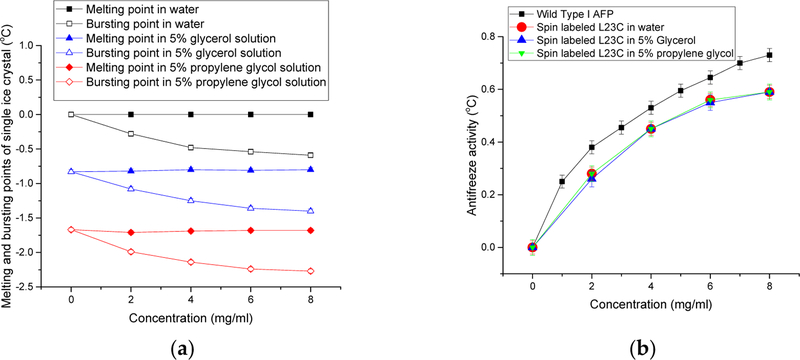
(**a**) Melting and bursting points of ice crystals in the spin labeled L23C water, 5% glycerol, and 5% propylene glycol solutions, respectively; and (**b**) antifreeze activities of the wild Type I AFP, and the spin-labeled L23C in water, 5% glycerol and 5% propylene glycol solutions, respectively.

Figure 2a shows the melting and bursting points of the ice crystals versus the concentrations of the spin-labeled L23C in water and the CPA solutions. (See the inset for the belongings of the curves.) It shows that the melting points of the ice crystals in these solutions did not vary with the increase of the concentrations of the spin-labeled AFP, but the bursting points decreased. Figure 2b shows the antifreeze activities of the spin-labeled AFP in these solutions at different concentrations. It shows that all the antifreeze activities of the spin labeled AFPs were the same at the same AFP concentrations in the three solutions although the melting points and bursting points of the ice crystals in these solutions with the same AFP concentrations were different. The results indicate that the antifreeze activities solely reflects the effect of the AFP on inhibiting the growth of ice crystals in the water and CPA solutions. It appears that the CPAs did not have a collaborative effect on the antifreeze activities. Thus, the experimental results show a two-step process to prevent ice growths in the AFP CPA solutions, i.e., (1) the thermodynamic freezing point depressions by glycerol and propylene glycol, respectively; and (2) inhibition to the growth of seed ice crystals by the AFPs.

For comparison, the antifreeze activities of wild Type I AFPs in water are also given in Figure 2b. The antifreeze activities of the spin-labeled L23C are a little lower than those of the wild type I AFPs. Although the spin label was attached to the non-ice binding residue, which faces the water phase, the antifreeze activities were affected to some extent. This is also true for a few other spin-labeled type I AFPs on other non-ice binding residues [34]. The experimental results indicate that the binding constant of an AFP to the ice surface is determined by both the ice-binding motifs and the properties of other residues. We believe that the binding of AFP to ice surface induced a Water-AFP-Ice (WAI) interphase between the ice phase and water phase [57]. The concentration of AFP in the interphase is much higher than that in the bulk water phase, thus the lowered freezing point of water in the interphase. The spin label is hydrophobic and bulkier than the replaced leucine side chain, which slightly changed the balance between the hydrophobicity and hydrophilicity of the wild type I AFP, resulting in a decreased spontaneity to form the WAI interphase, and, thus, less antifreeze activity. This behavior shows the nature of thermodynamic equilibrium of AFP’s ice binding. Otherwise, if the binding of AFP to ice surface were irreversible, all kinds of AFPs would have the same antifreeze activity, which would disagree with literature on this subject. Our theoretical model is the ice surface adsorption enhanced the colligative effect of AFPs in the WAI interphase [57]. An AFP’s antifreeze activity is determined by the decreased Gibbs energy of the WAI interphase leading to the decreased freezing point in the interphase. A larger binding constant makes the AFP’s concentration higher in the WAI interphase; thus a lowered freezing point of water in the WAI interphase. The WAI interphase acts as a thermodynamic barrier between the ice phase and the water phase. The ice phase was in thermal equilibrium with the WAI interphase while the water phase was in super-cooled condition.

### 3.2. EPR Results of the Spin Labeled Type I AFP in the CPA Solutions

**Figure 3.**
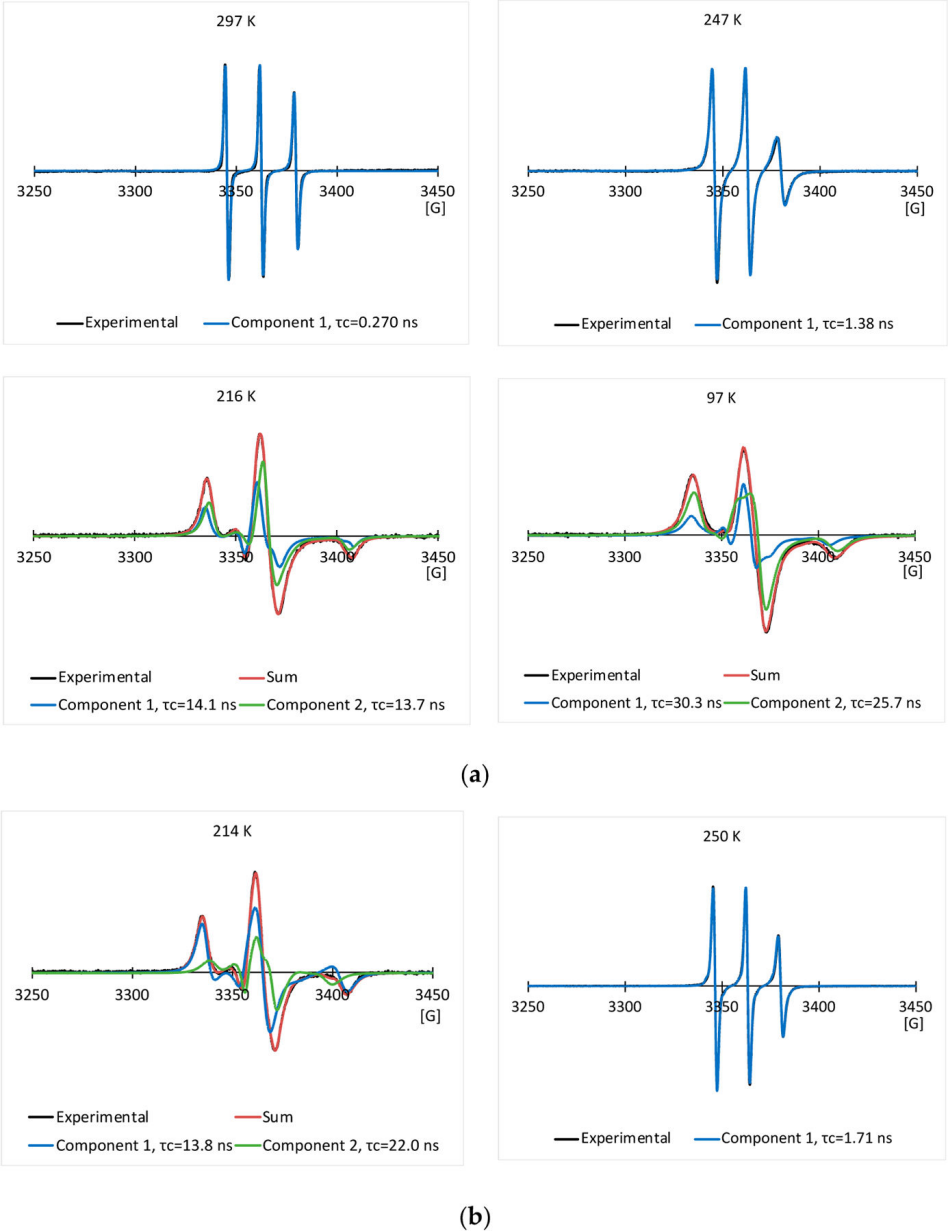
Typical experimental EPR spectra and their simulated EPR spectra of the 2.0 mg/mL spin labeled L23C in the 5% glycerol solution through the freezing process (**a**), and melting process (**b**), respectively.

**Figure 4.**
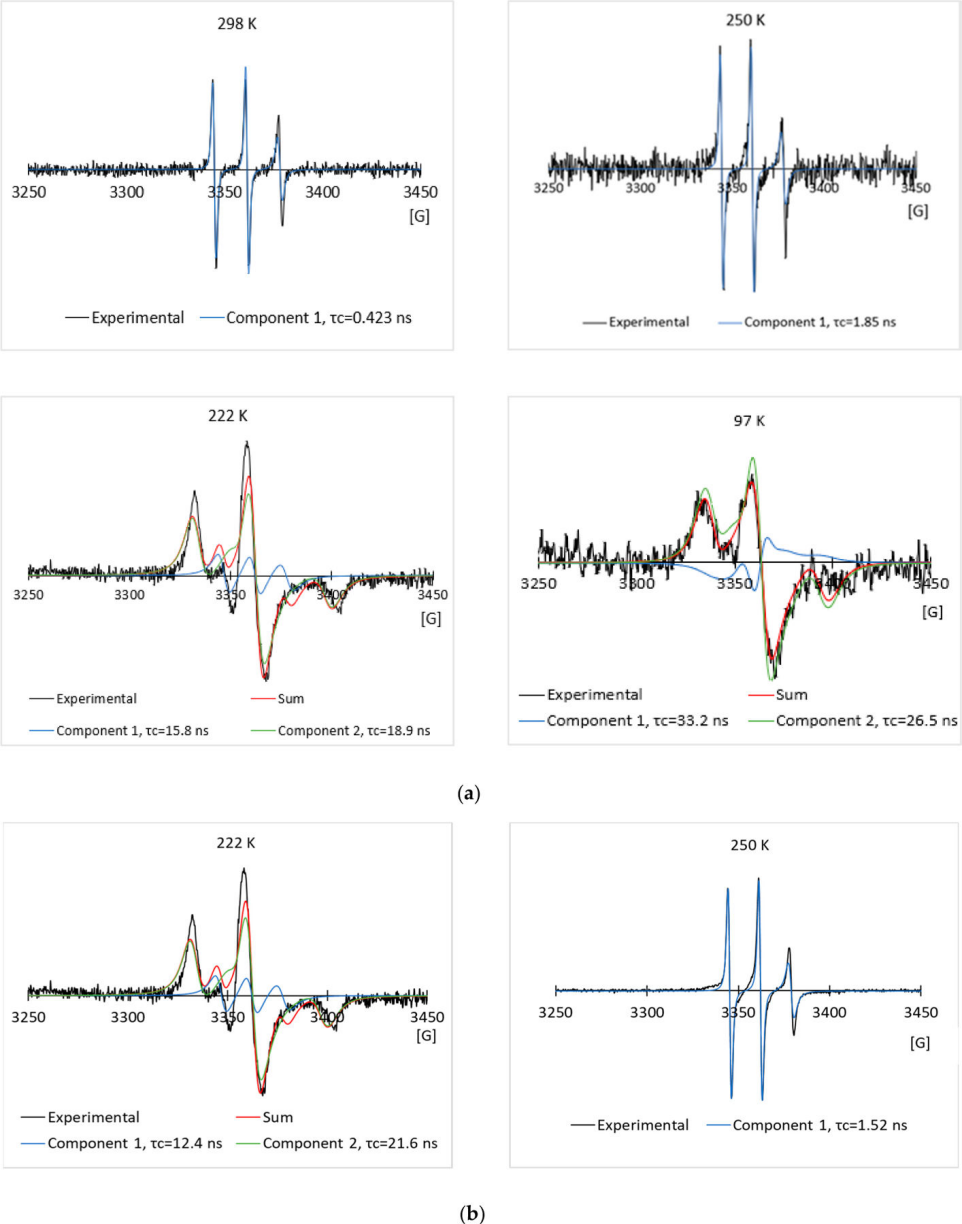
Typical experimental EPR spectra, and their simulated EPR spectra of the 2.0 mg/mL spin labeled L23C in the 5% propylene glycol solution through the freezing process (**a**), and melting process (**b**), respectively.

Figure 3a,b show some typical experimental EPR spectra, and their simulated EPR spectra of the 2.0 mg/mL spin labeled L23C in the 5% glycerol solution through the freezing process, and melting process, respectively. The temperatures and corresponding correlation times are given in the inset of the spectra. The nitroxide radical has a triplet EPR spectrum, due to the coupling of the electron spin with the ^14^N nucleus spin. Narrow lineshape shows free tumbling of the molecule in a solution. With the decrease in temperature, the lineshape became broader, showing the restricted motion of the spin labels, due to the freezing of the solution. Spectral simulation allows us to obtain the rotational correlation time (τ_c_) for the motion of the spin labels, and also possibly the number of components included in the spectrum. For a random molecular tumbling, τ_c_ roughly corresponds to the average time for a molecule to progress through one radian. Here, the correlation time primarily represents that of chemical bond rotation of the MSL spin label, while the tumbling of the whole spin labeled AFP was much slower, especially after the solutions were frozen. Figure 4a,b show those of the 2.0 mg/mL spin labeled L23C in the 5% propylene glycol solution. The simulations show that one EPR spectral component existed at higher temperatures (> ~245 K), and minimum two components could fit the spectra well at lower temperatures for both the freezing and melting processes.

**Figure 5.**
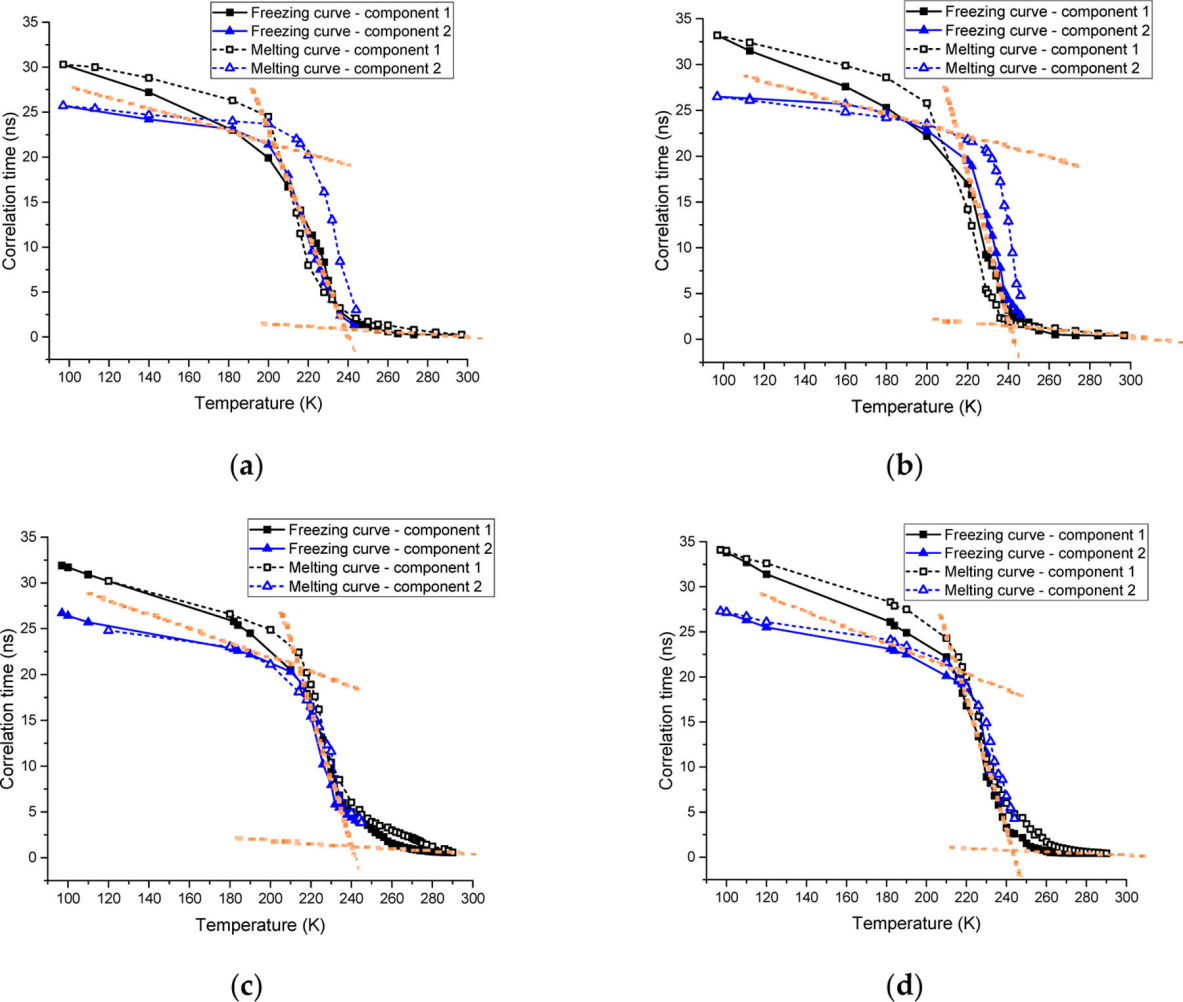
(**a**) Curves of correlation times (τ_c_) of the 2.0 mg/mL spin labeled L23C in the 5% glycerol solution versus temperature through the freezing process, and melting process; (see the inset in the Figure.); (**b**) those of the 2.0 mg/mL spin labeled L23C in the 5% propylene glycol solution; (**c**) those of the 2.0 mg/mL spin labeled A17C in the 5% glycerol solution; and (**d**) those of the 2.0 mg/mL spin labeled A17C in the 5% propylene glycol solution.

Figure 5a shows the curves of correlation times (τ_c_) of the 2.0 mg/mL spin labeled L23C in the 5% glycerol solution versus temperature (T) through the freezing process and melting process, respectively. (See the inset for belongings of the curves.) The correlation times increased with the decrease in temperature in the freezing process, showing the decreased kinetic energy of the chemical bond rotation of the spin label, and also steric hindrance of the chemical bond rotation, due to the freezing of the surrounding fluid at low temperatures. The slope of the curve changed rapidly between 250.0 K and 230.0 K, and meanwhile, more than one component of the simulated EPR spectra started appearing at 243.0 K, which shows that the surroundings of the MSL spin labels began to freeze at temperatures below 245 K. To show the sharp changes of the curves more visually, dotted red lines were drawn through the average values of the three near linear regions. The two-component model allowed us to fit the EPR spectra well at low temperatures, while the one-component model yielded poor fitting. However, more-component models would also allow us to fit the EPR spectra at the low temperatures. Thus, the two-component model should be understood as the distribution of correlation times between the shorter one and the longer one of the two components. The correlation times increased rapidly from 230.0 to 210.0 K, and then turned to increase slowly until the lowest experimental temperature of 97.0 K. The steep slope ranging from 230.0 to 210.0 K indicates that freezing of the surroundings around the MSL spin labels progressed quickly, while the slow increase below 200 K indicates that the surroundings of the MSL spin labels became solidified although hardness of the frozen solution continued to increase until 97 K. As shown in Figure 5b, curves of the correlation times versus temperature for the spin labeled L23C in the propylene glycol solution are similar to those of the glycerol solution. The freezing processes monitored by the spin labels attached to the L23C in the CPA solutions were quite different from that in water [34]. The latter showed a sharp local phase transition at 251.6 K. However, no sharp phase transitions were observed in the CPA solutions. The freezing curves in Figure 5a,b illustrate that the eutectic points decreased with the decrease in temperature, due to the increased amount of ice, which made the concentrations of the CPAs in the solution phases higher at lower temperatures. Distributions of the correlation times during the freezing processes indicate the heterogeneous environments surrounding different spin labels although not significant above 200 K. The spin labeled L23C bound to ice surfaces, while the spin labels interacted with water and glycerol, or propylene glycol in the WAI interphase during the freezing process. The thickness of the WAI interphase could have a distribution, which affected the mobility of the spin labels in a range. The solidified matrices below 200 K made the spin label’s surroundings more anisotropic, thus broader distributions of the correlation times.

Curves of the correlation times versus temperature through the melting processes are similar to those of the freezing processes, but with broader distributions of correlation times, and larger average correlation times than those of the freezing curves for both of the CPA solutions. Recall that a larger correlation time means less mobility of the spin label. Therefore, it appears that the spin labeled L23C inhibited the ice growth during the freezing processes of the CPA solutions, thus shorter correlation times compared with those during the melting processes at the same temperatures. In other words, the melting happened at closer thermal equilibrium temperatures. The broader distributions of the correlation times during the melting processes could indicate that the melting points (eutectic points) are not quite spatially homogeneous in the samples. As compared with the inactive spin labeled A17C below, this phenomenon can be related to the ice surface binding of spin labeled L23C possibly during the freezing processes.

**Figure 6.**
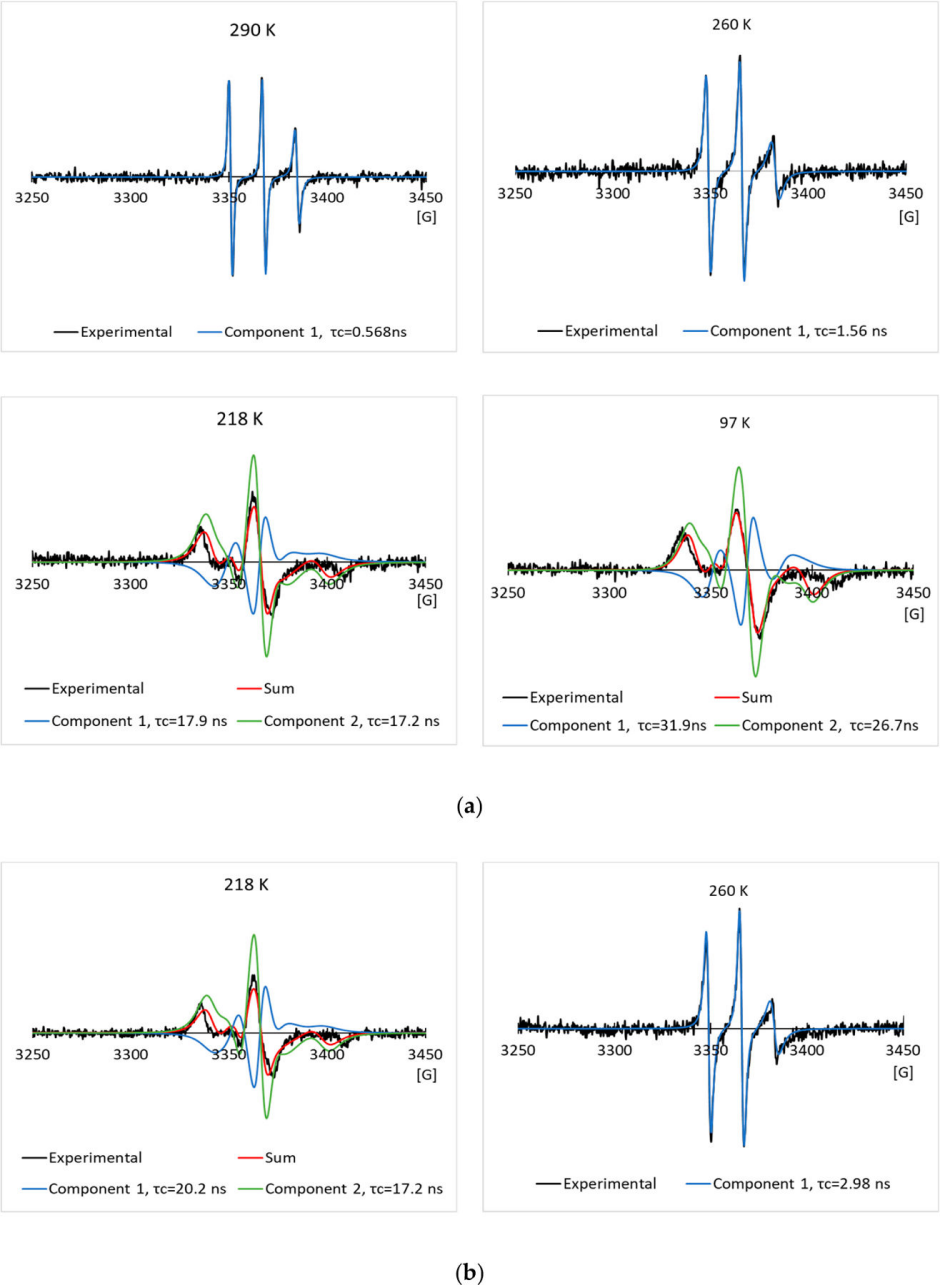
Typical experimental EPR spectra, and their simulated EPR spectra of the 2.0 mg/mL spin labeled A17C in the 5% glycerol solution in the freezing process (**a**), and melting process (**b**), respectively.

**Figure 7.**
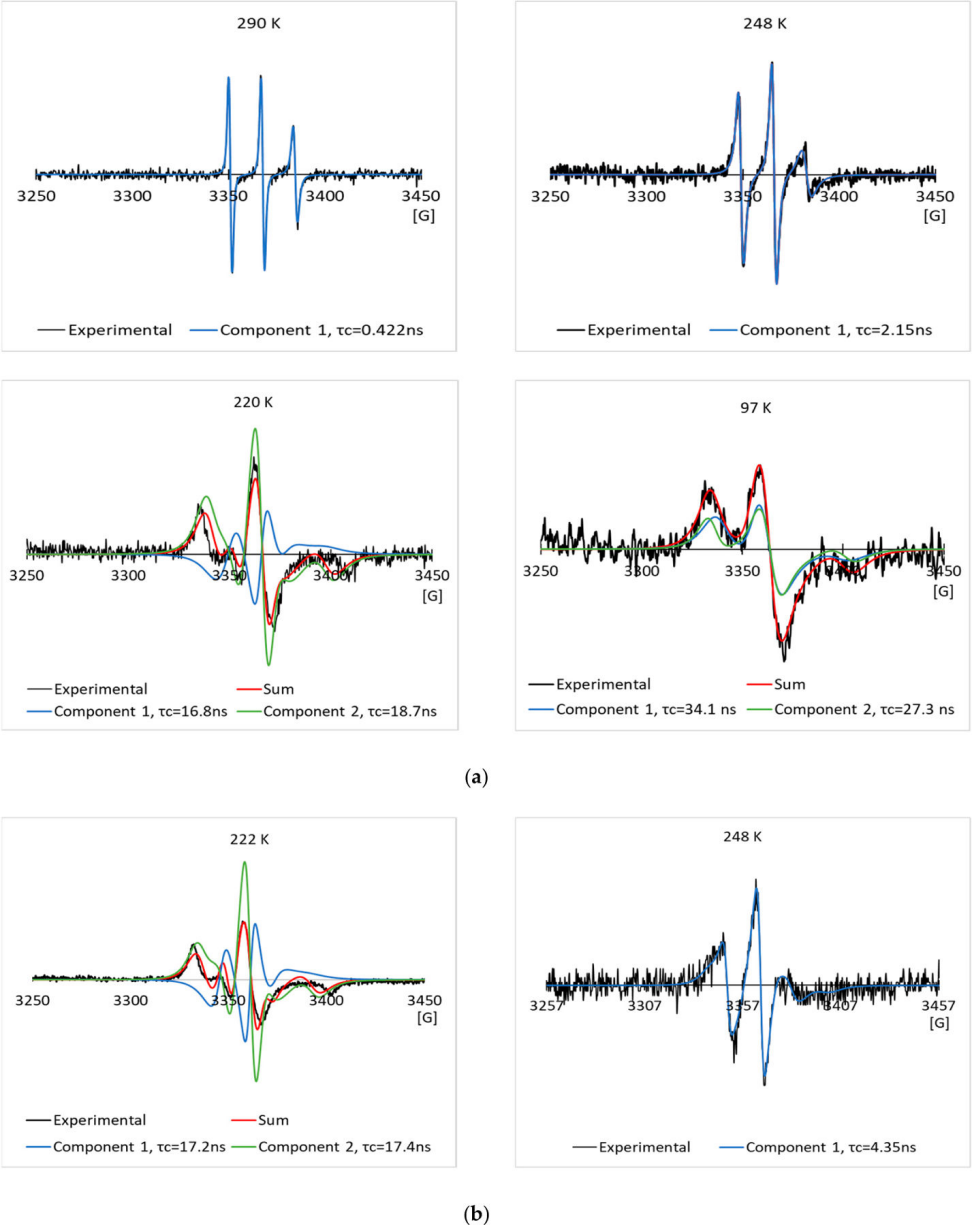
Typical experimental EPR spectra, and their simulated EPR spectra of the 2.0 mg/mL spin labeled A17C in the 5% propylene glycol solution in the freezing process (**a**), and melting process (**b**), respectively.

To compare with the negative control, we have run the EPR experiments on the 2.0 mg/mL spin labeled A17C in the 5% glycerol solution, and the 5% propylene glycol solution through the freezing and melting processes. The experimental and simulated EPR spectra are shown in Figure 6a,b and Figure 7a,b respectively. As in the L23C CPA solutions, the two-component model was used to simulate the EPR spectra at low temperatures. Curves of the corresponding correlation times versus temperatures are shown in Figure 5c,d for the A17C in the glycerol, and propylene glycol solutions, respectively. Different from the spin labeled L23C CAP solutions, the freezing curves and melting curves are quite close, showing that the inactive spin labeled A17C did not bind to ice surfaces. As shown by the crossing points of the dotted red lines between 250 K and 230 K, the freezing point of the A17C CPA solutions were about five degrees higher than the L23C CPA solutions, further showing that the active spin labeled L23C inhibited the ice growths during the freezing process. Another noticeable phenomenon is that the correlation times of the A17C CPA solutions decreased slower during the melting processes at temperatures higher than 240 K, which can also be observed on the corresponding EPR spectra in Figure 6 (260 K) and Figure 7 (248 K). The broader EPR linewidths during the melting processes indicate slower rotational motions, or longer correlation times of the spin labels. This phenomenon shows that the spin labeled A17C peptides did not bind to ice surfaces, but were instead pushed to aggregate due to the freezing of the solutions. Thus, the dissolution process of the spin labeled A17C peptides could not be as fast as the individually ice surface bound spin labeled L23C peptides during the melting process. Results of the control experiments further indicate that the spin labeled L23C bound to ice surfaces during the frozen process and in the frozen CPA matrices.

## 4. Conclusions

Both results of cryo-photo microscopy and EPR spectral analysis indicate that the spin labeled AFP bound to ice surfaces at all temperatures as long as ice formed in the CPA solutions. A two-step process, i.e., (1) thermodynamic freezing point depression by the CPAs, and (2) inhibition on the growth of ice crystals by the AFP, occurred to prevent the formation of bulk ice in the CPA solutions at low temperatures. The spin labeled AFP bound to ice surfaces during the bulk freezing process, and appeared to inhibit the ice growth in the CPA solutions at low temperatures. The ice surface bound AFP in the frozen bulk solutions could also prevent ice recrystallization during the melting process. We, therefore, conclude that AFPs are potentially useful as an active ingredient to make CPA solutions for preventing cryo-damages of living cells, organs and tissues, and for industrial applications.

## Supplementary Material

Supplemental files
