# Supplementary material for: Effect of Type I Antifreeze Proteins on the Freezing and Melting Processes of Cryoprotective Solutions Studied by Site-Directed Spin Labeling Technique
Source: Crystals (Basel). Author manuscript; Available in PMC 2020 Nov 20. (PMC7678753; doi:10.3390/cryst9070352)
Supplement: Supplemental files [file NIHMS1046303-supplement-Supplemental_files.pdf]

Supporting Materials

Figures 1-5 show the MALDI TOF spectra of the cysteine substituted HPLC6 mutants and their MSL spin labeled ones. The MALDI TOF spectra show the spin labeled products of these mutants.

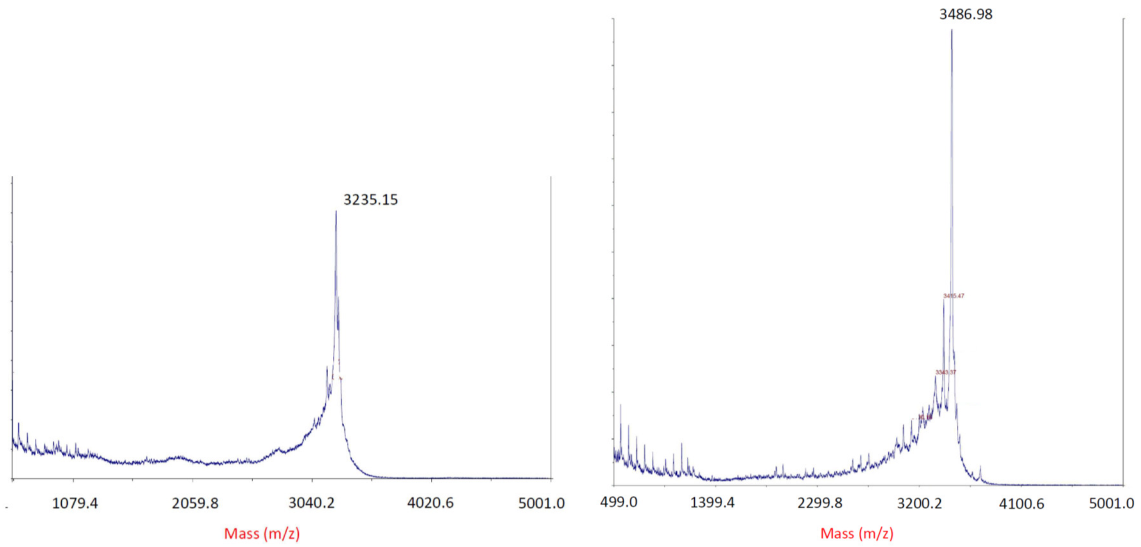

Figure 1. (Left) MALDI TOF spectrum of HPLC6 L23C peptides showing molar mass of 3235.15 Da; (Right) MALDI TOF spectrum of MSL spin labeled HPLC6 L23C peptides showing molar mass of 3486.98 Da.

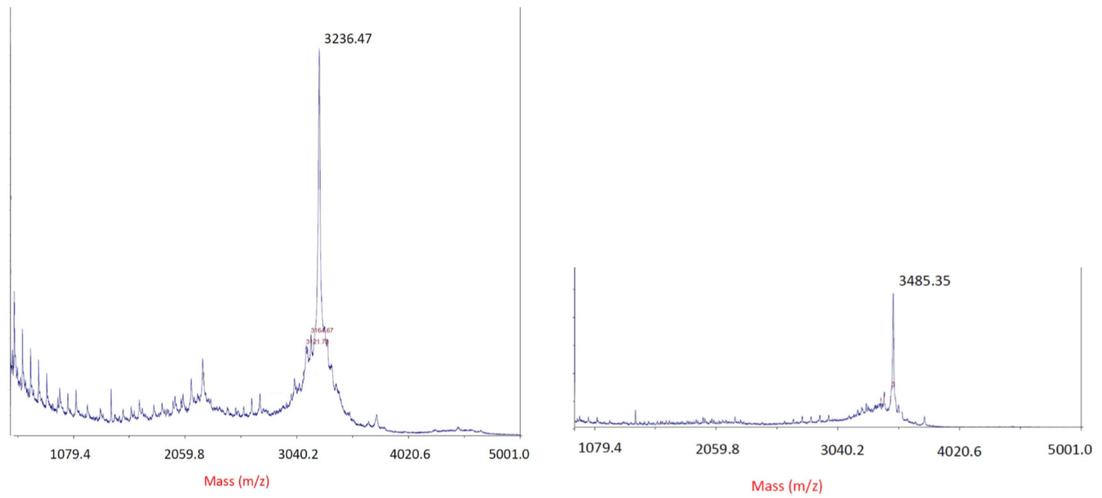

Figure 2. (Left) MALDI TOF spectrum of HPLC6-L12C peptides showing molar mass of 3236.47 Da; (Right) MALDI TOF spectrum of MSL spin labeled HPLC6-L12C peptides showing molar mass of 3485.35 Da.

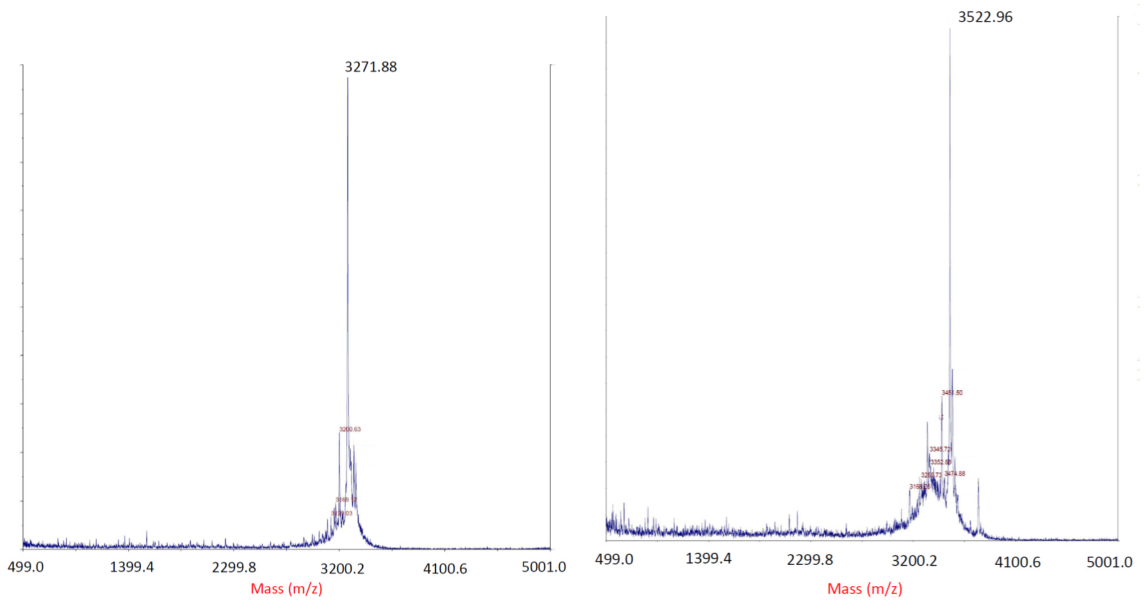

Figure 3. (Left) MALDI TOF spectrum of HPLC6 A20C peptides showing molar mass of 3271.88 Da; (Right) MALDI TOF spectrum of MSL spin labeled HPLC6 A20C peptides showing molar mass of 3522.96 Da.

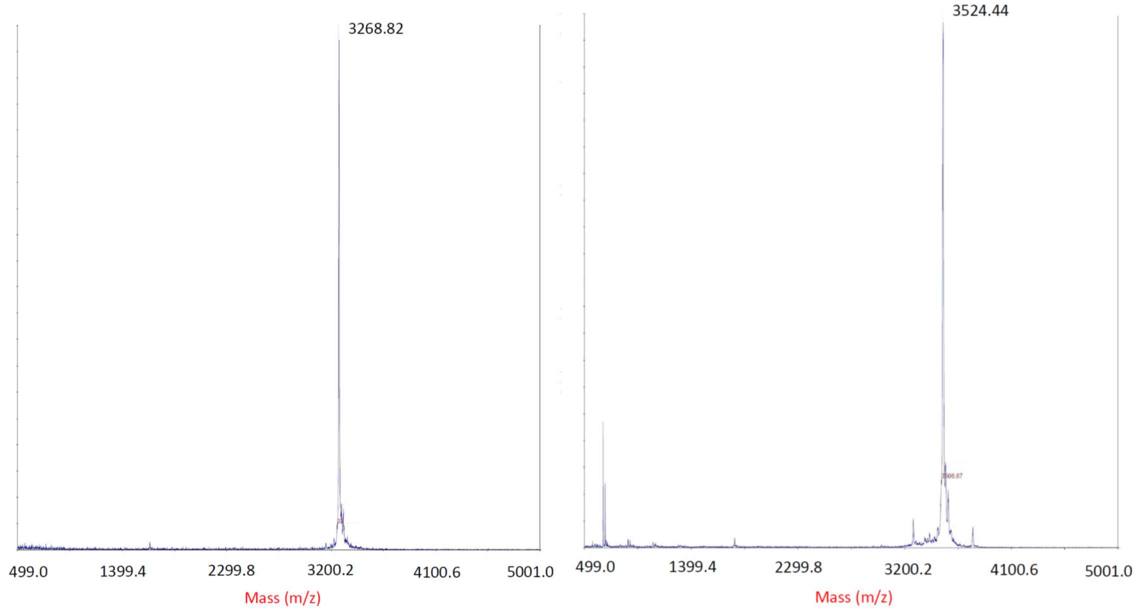

Figure 4. (Left) MALDI TOF spectrum of HPLC6 A11C peptides showing molar mass of 3268.82 Da; (Right) MALDI TOF spectrum of MSL spin labeled HPLC6 A11C peptides showing molar mass of 3524.44 Da.

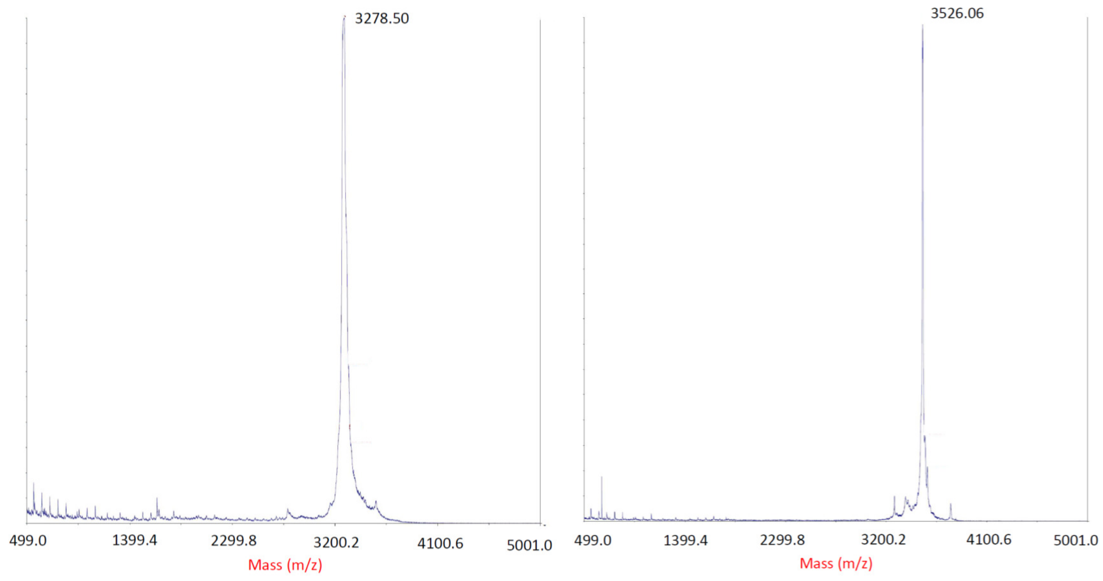

Figure 5. (Left) MALDI TOF spectrum of HPLC6 A17C peptides showing molar mass of 3278.50 Da; (Right) MALDI TOF spectrum of MSL spin labeled HPLC6 A17C peptides showing molar mass of 3526.06 Da.

Figures 6- 11 show the experimental VT EPR spectra of MSL spin labelled HPLC6 mutants.

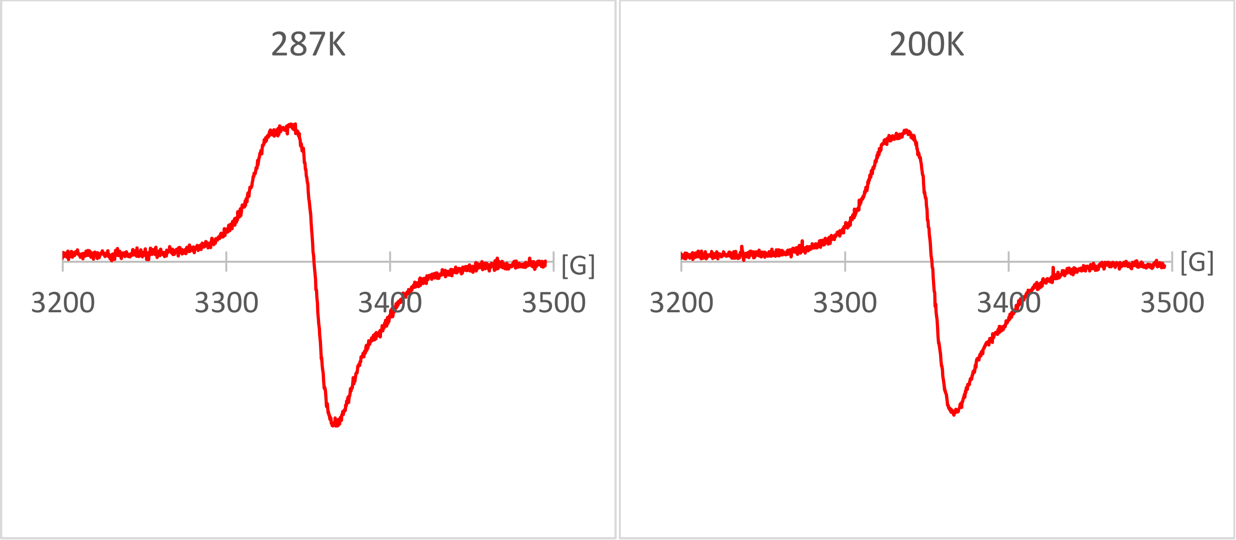

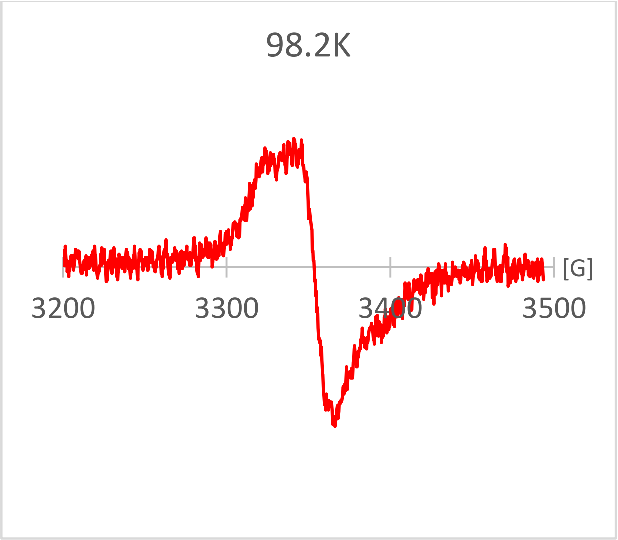

(a) Spin labeled HPLC6 L23C peptides

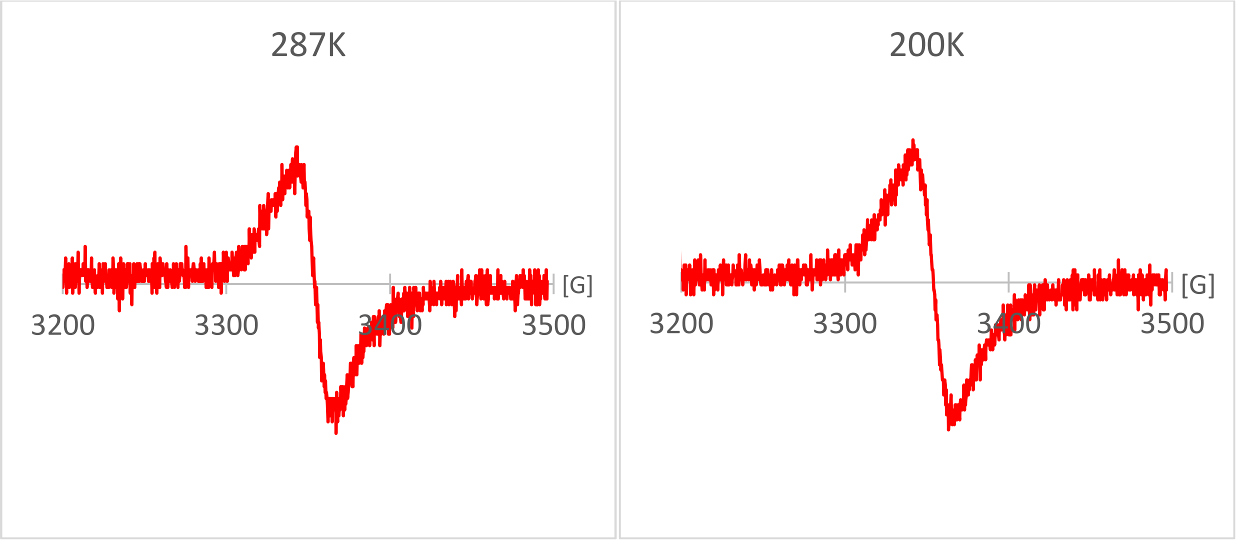

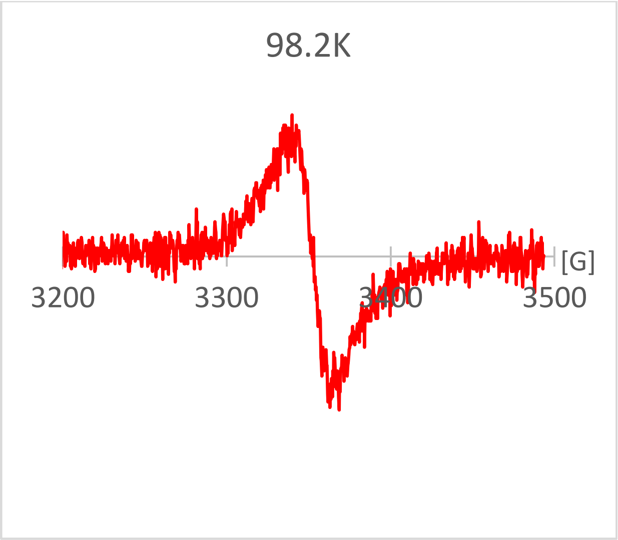

(b) Spin labeled HPLC6 L12C peptides

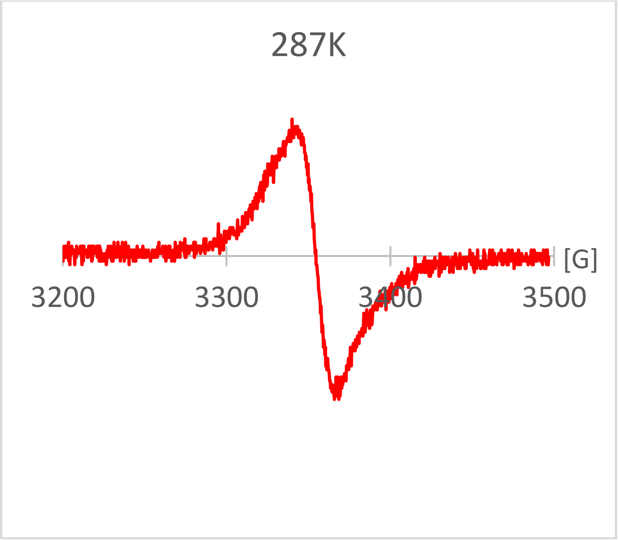

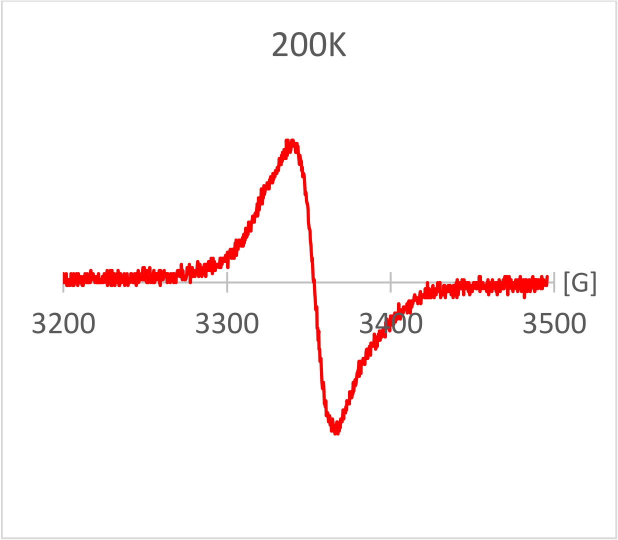

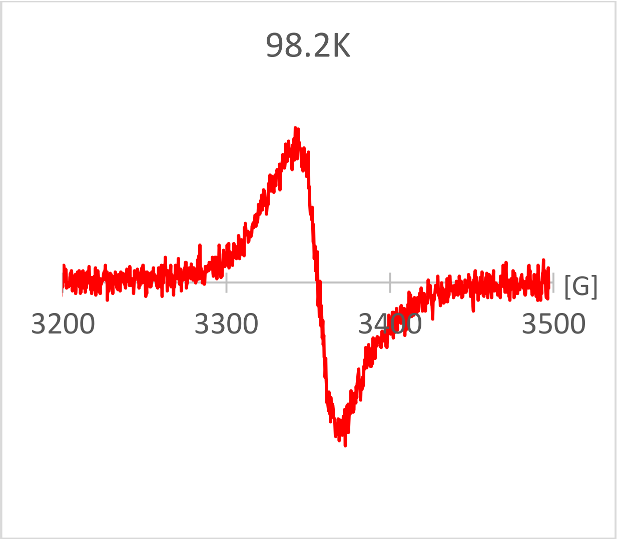

(c) Spin labeled HPLC6 A20C peptides

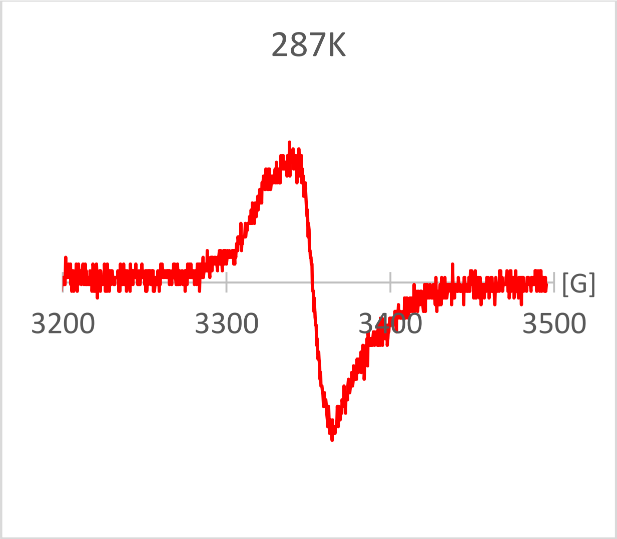

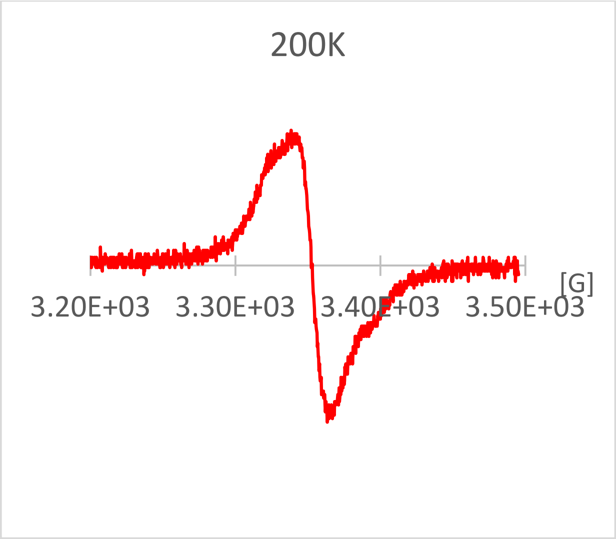

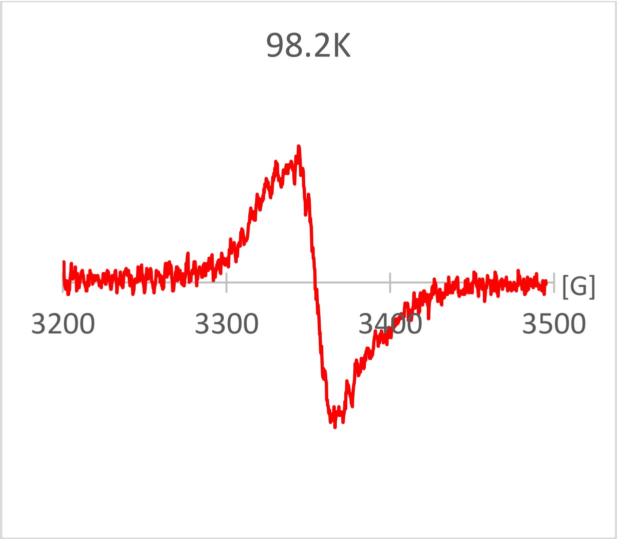

(d) Spin Labeled HPLC6 A11C peptides

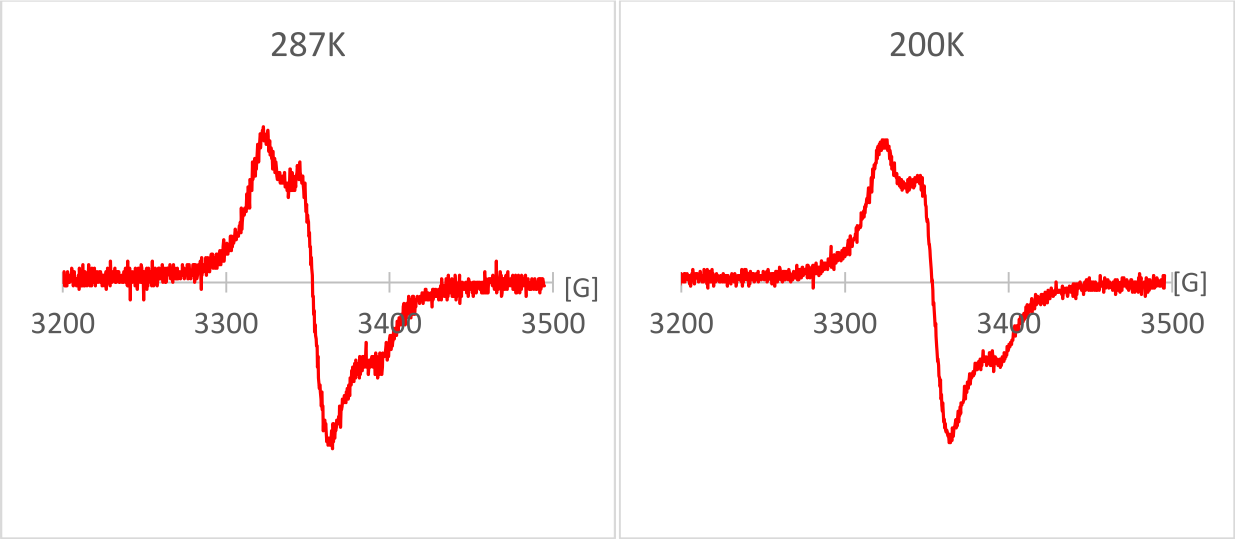

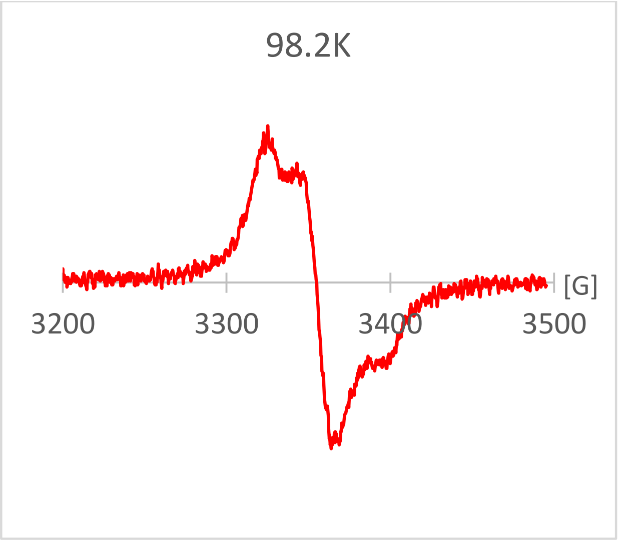

(e) Spin labeled HPLC6 A17C peptides

Figure 6. VT EPR spectra of MSL spin labeled HPLC6 peptide solid powders at different temperatures through the cooling process. Those through the heating process look the same.

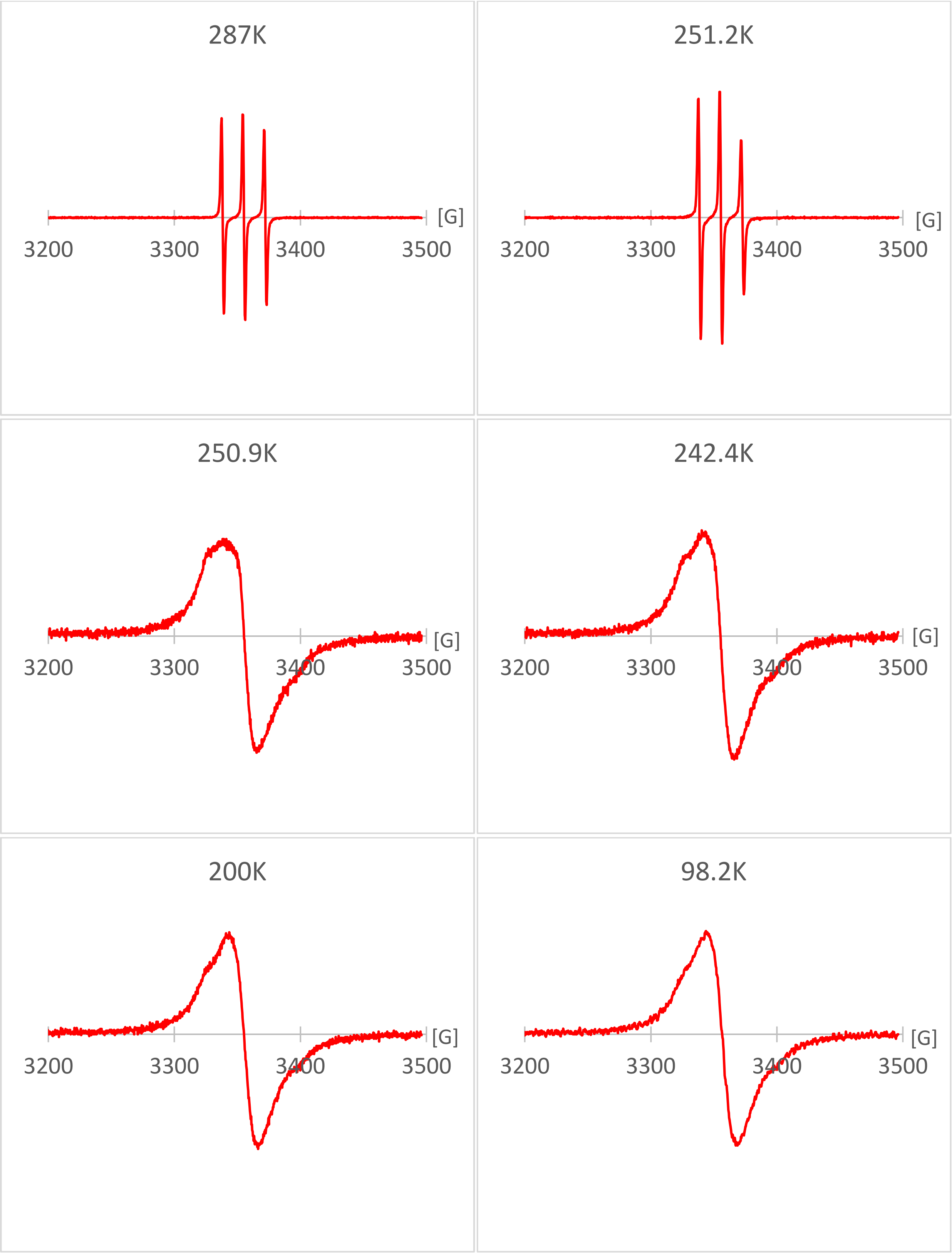

(a) Through cooling process

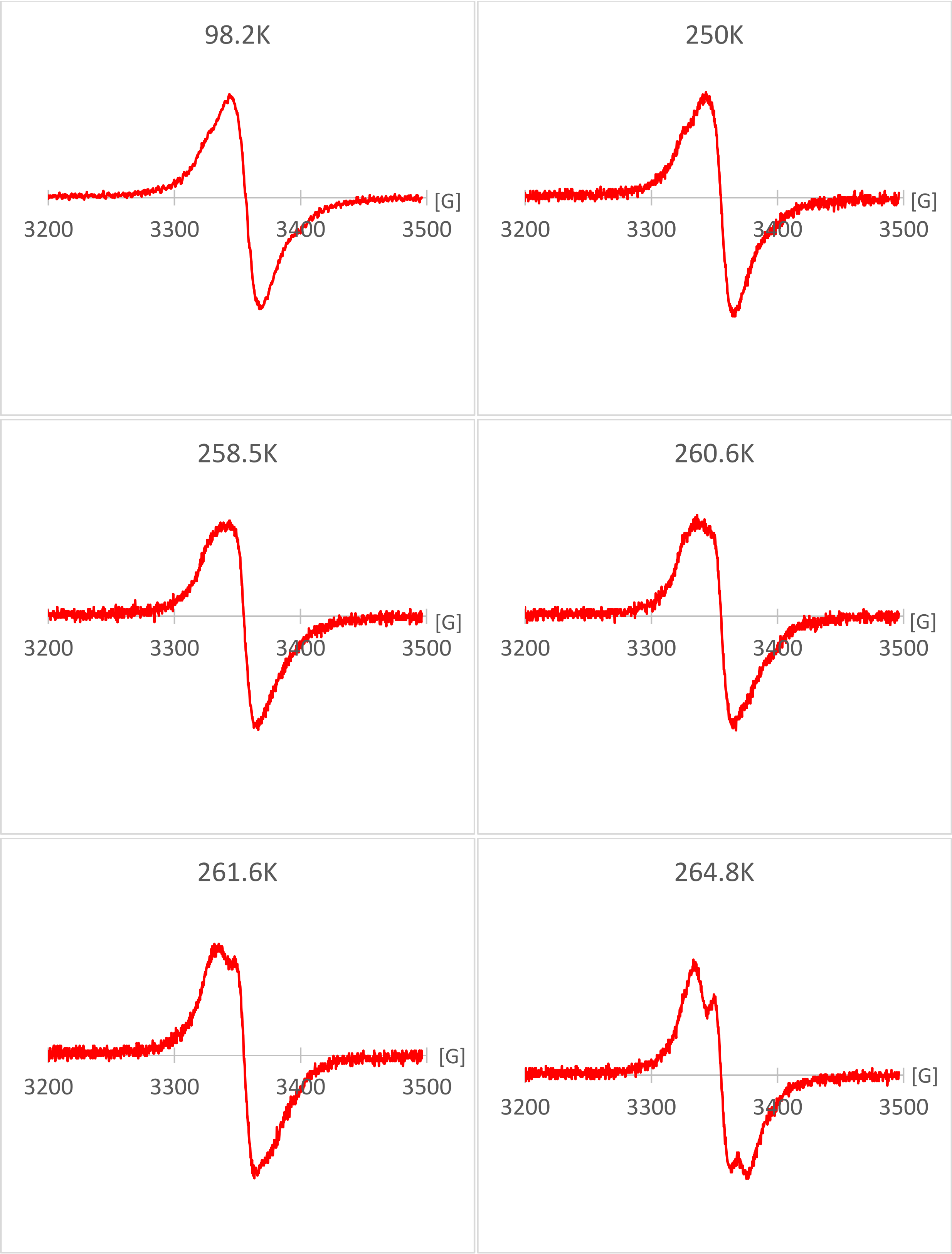
98.2K
250K
258.5K
260.6K
261.6K
264.8K
3200
3300
3400
3500
[G]

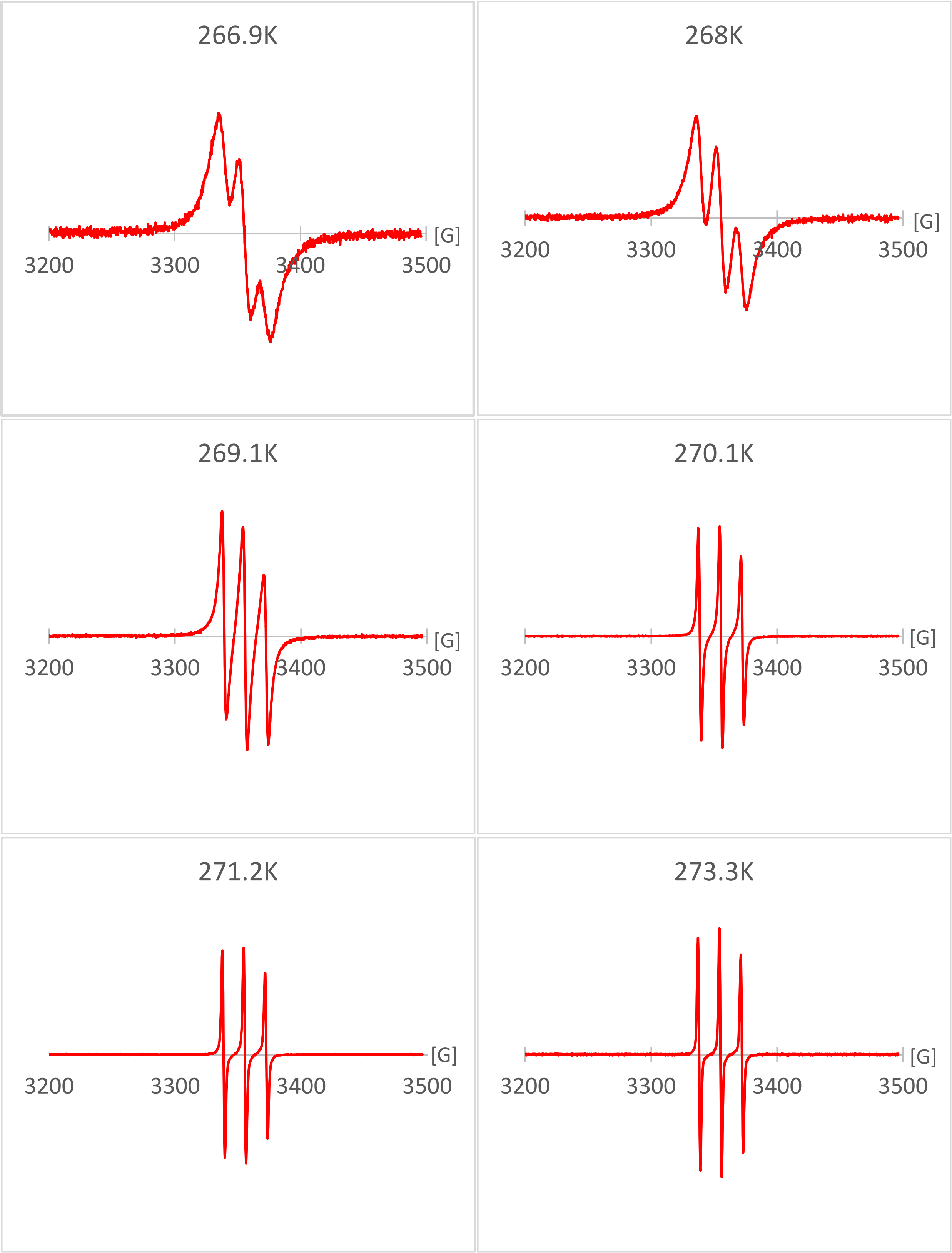

(b) Through heating process

Figure 7. VT EPR spectra of MSL spin labeled HPLC6 L12C water solution through the cooling process (a) and through the heating process (b).

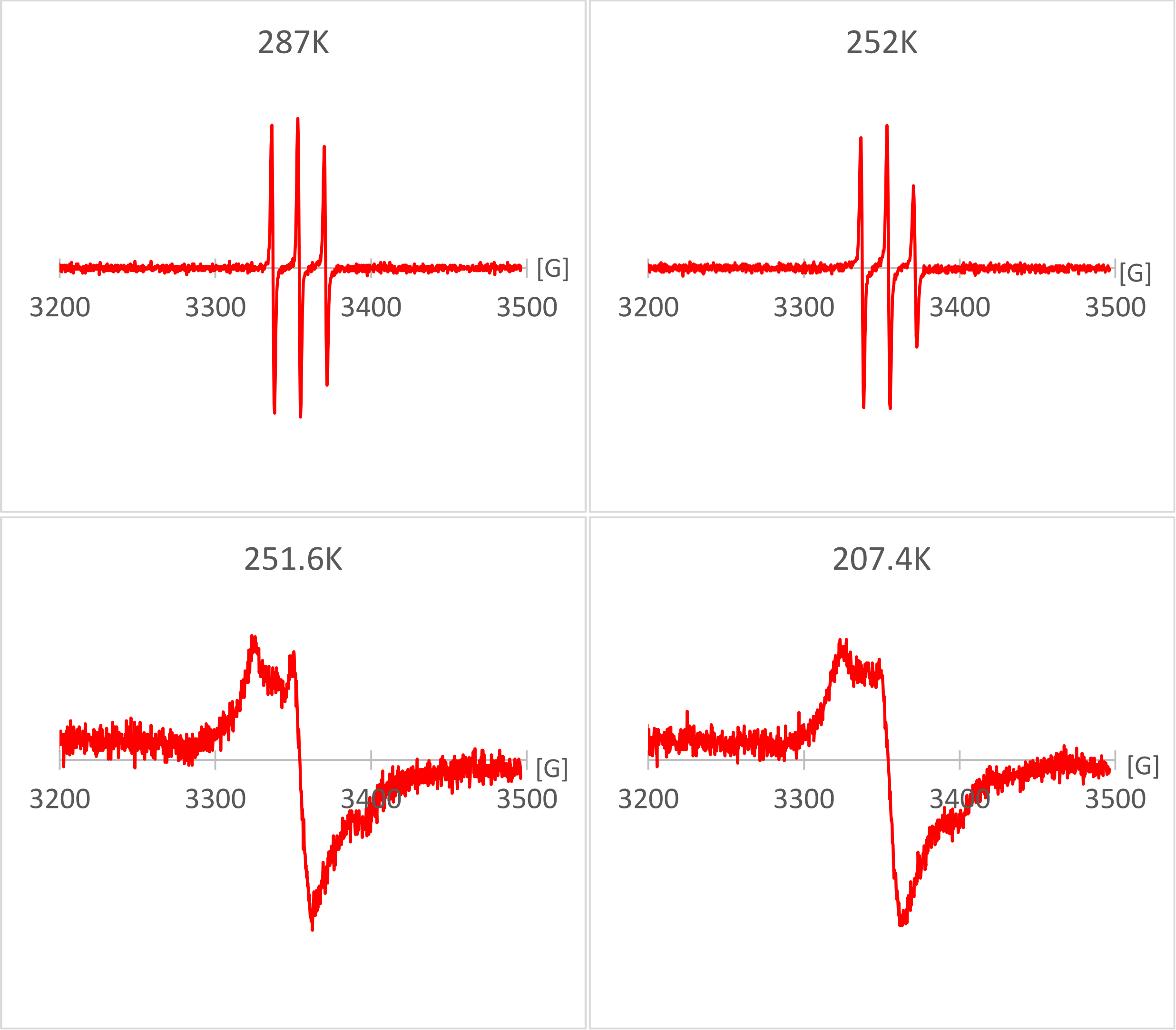

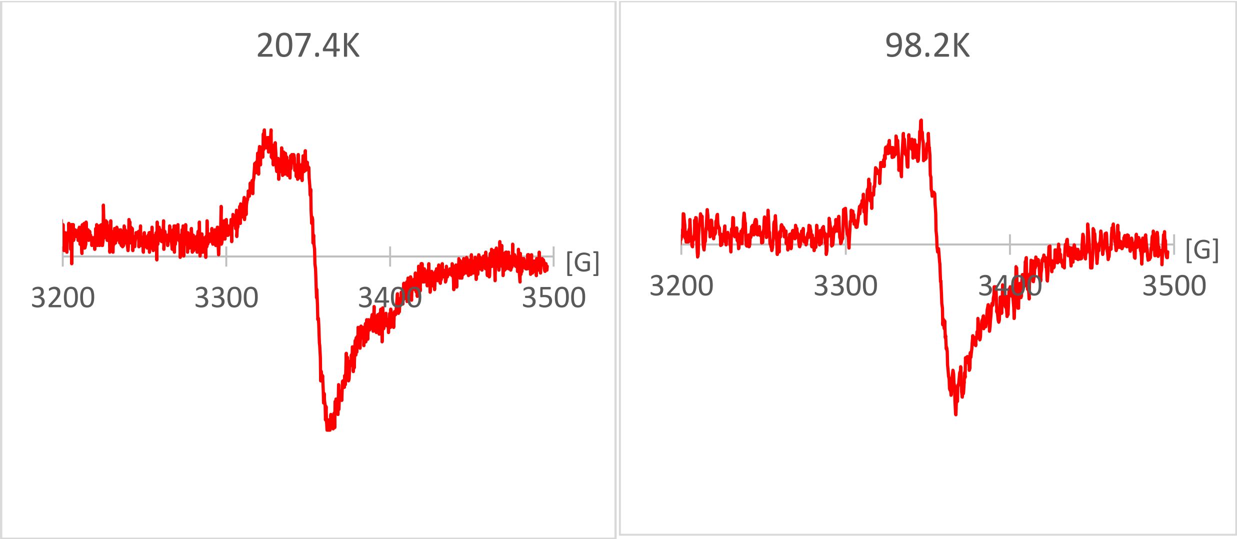

(a) Through cooling process

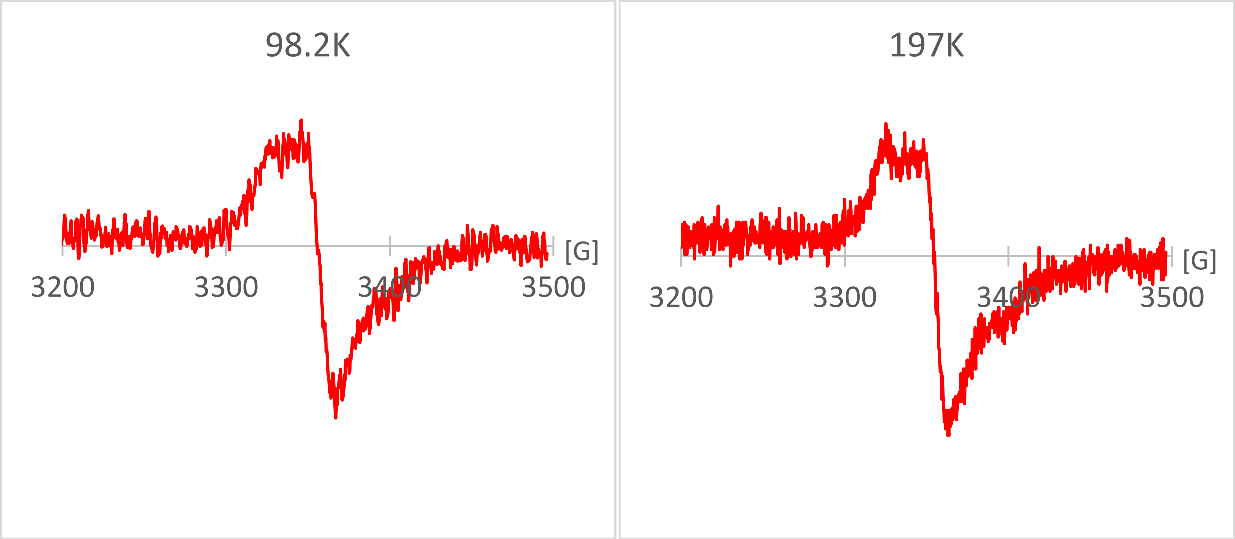

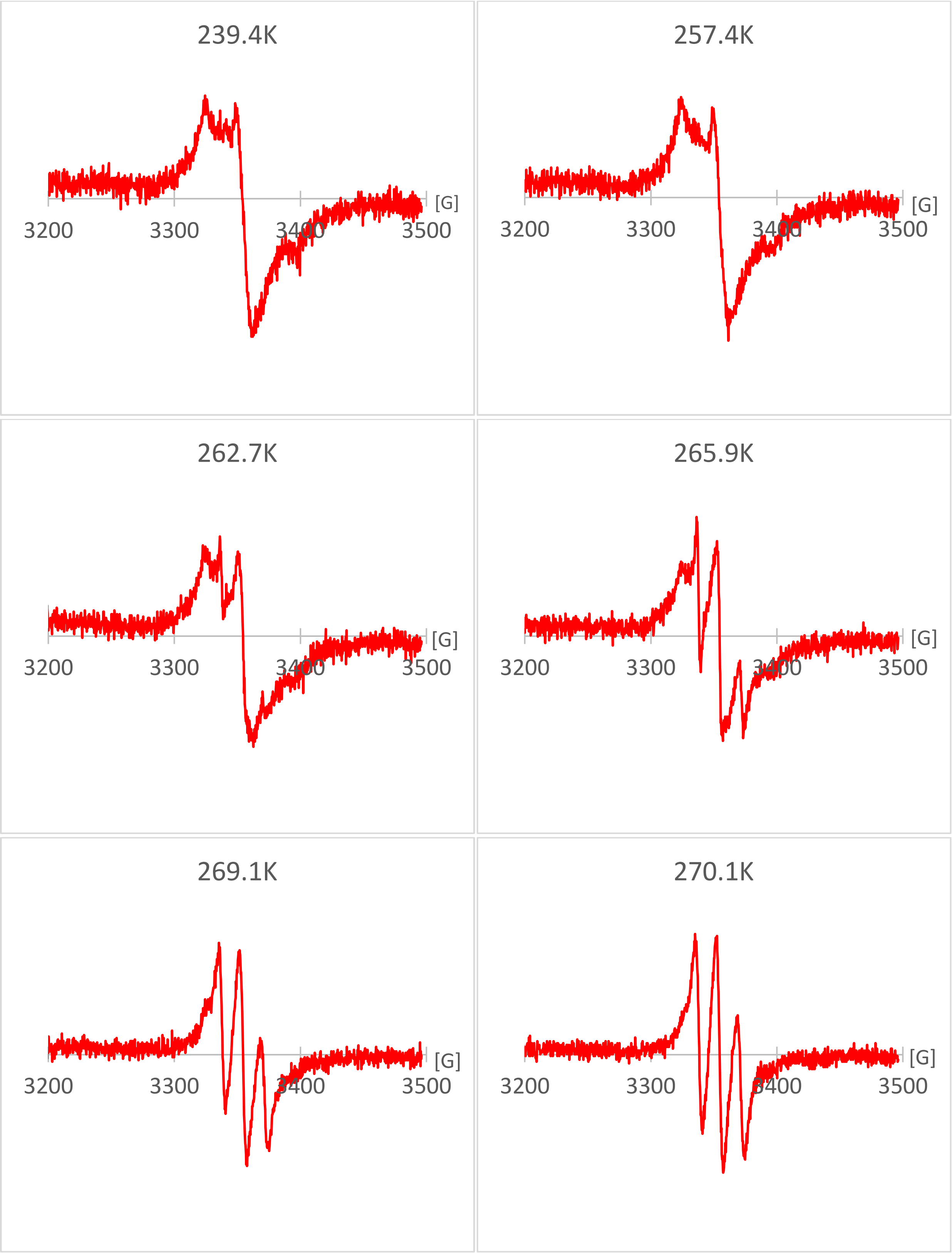
239.4K
257.4K
262.7K
265.9K
269.1K
270.1K
3200
3300
3400
3500
[G]

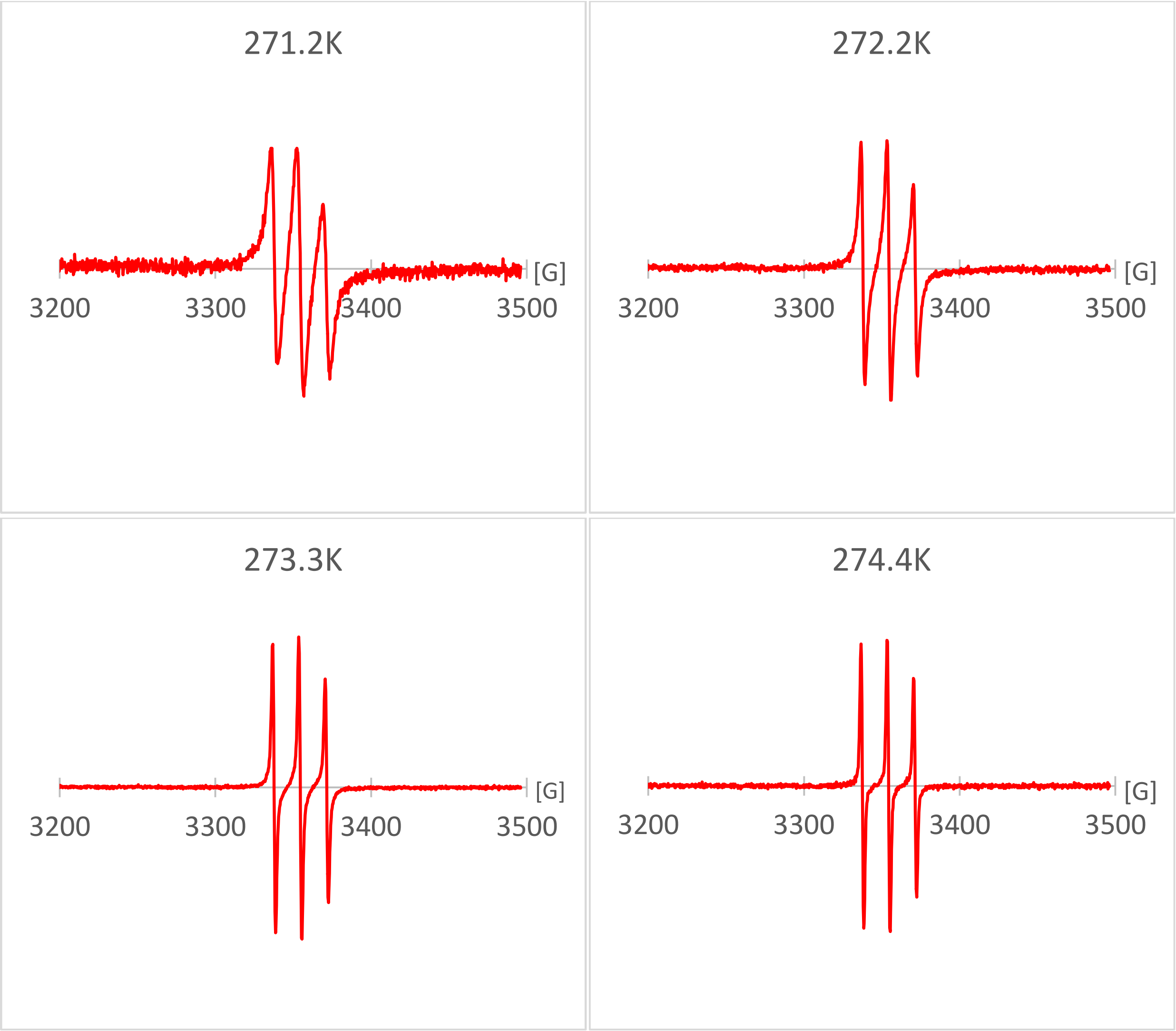

(b) Through heating process

Figure 8. VT EPR spectra of MSL spin labeled HPLC6 L23C water solution through the cooling process (a) and through the heating process (b).

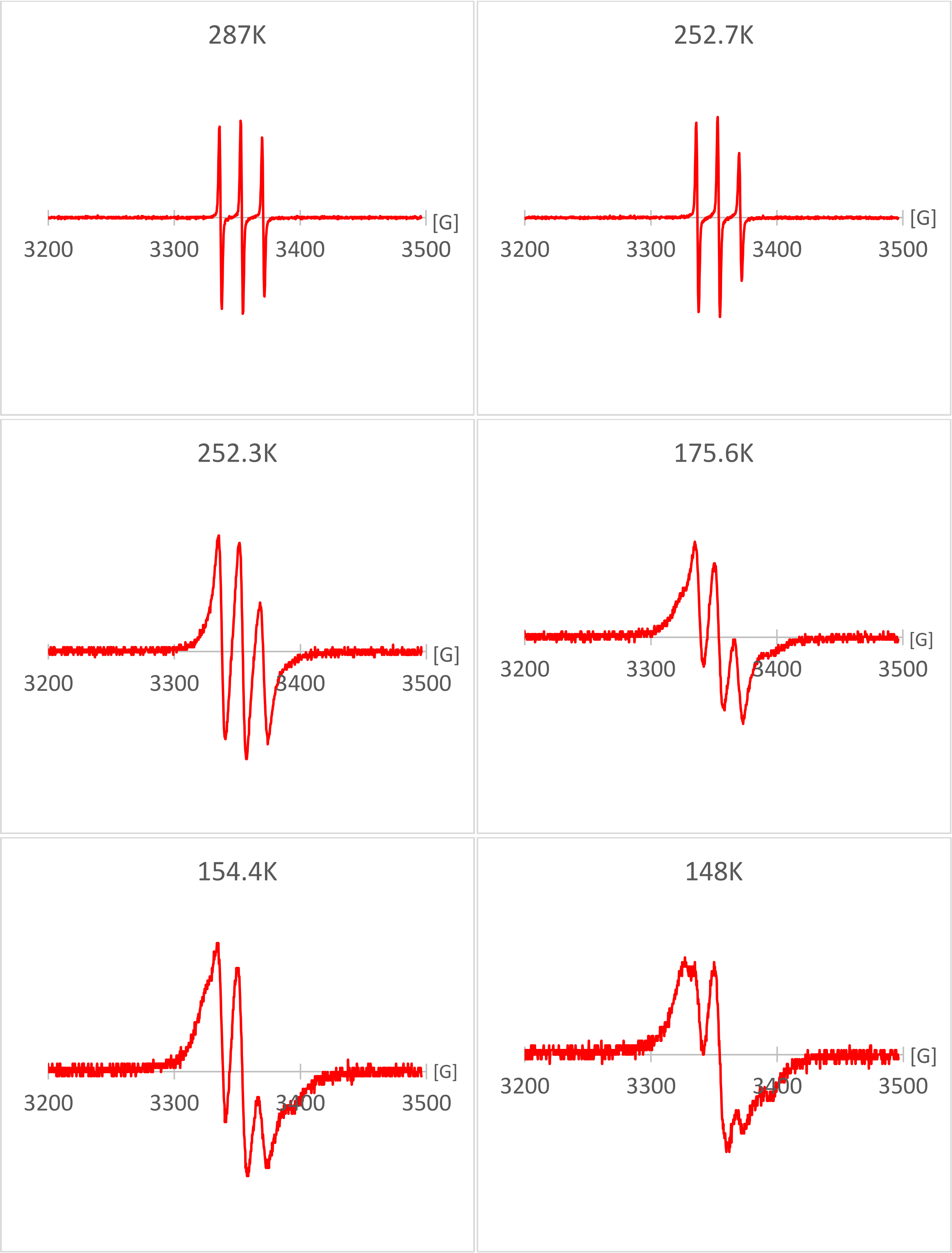
287K
3200
3300
3400
3500
[G]
252.7K
3200
3300
3400
3500
[G]
252.3K
3200
3300
3400
3500
[G]
175.6K
3200
3300
3400
3500
[G]
154.4K
3200
3300
3400
3500
[G]
148K
3200
3300
3400
3500
[G]

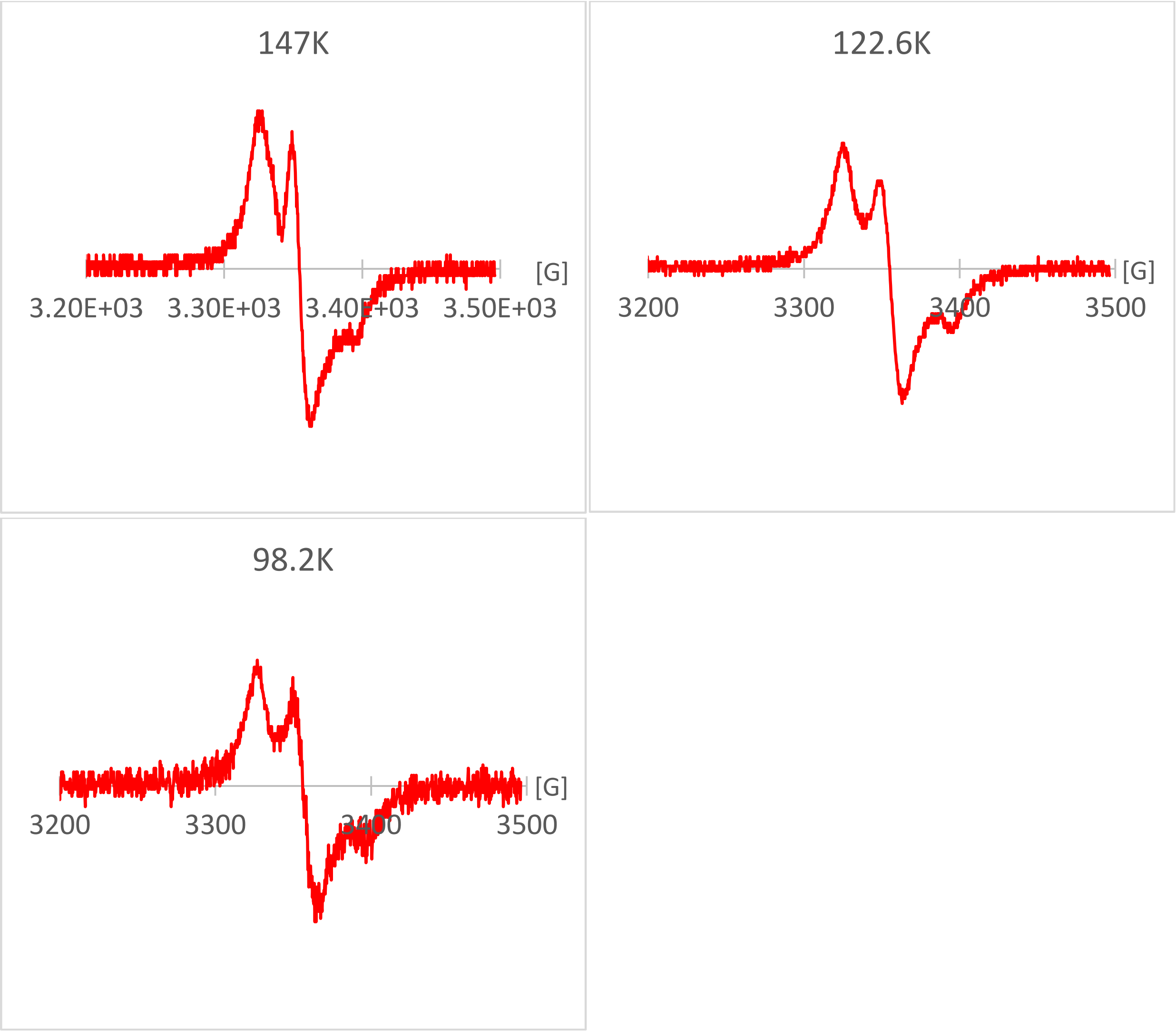

(a) Through cooling process

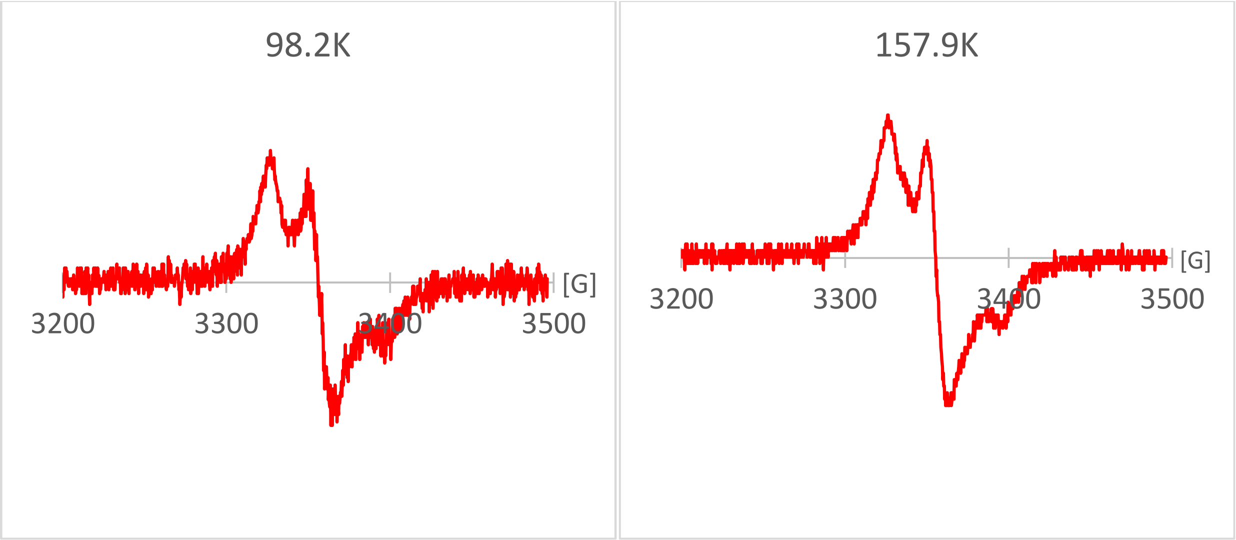

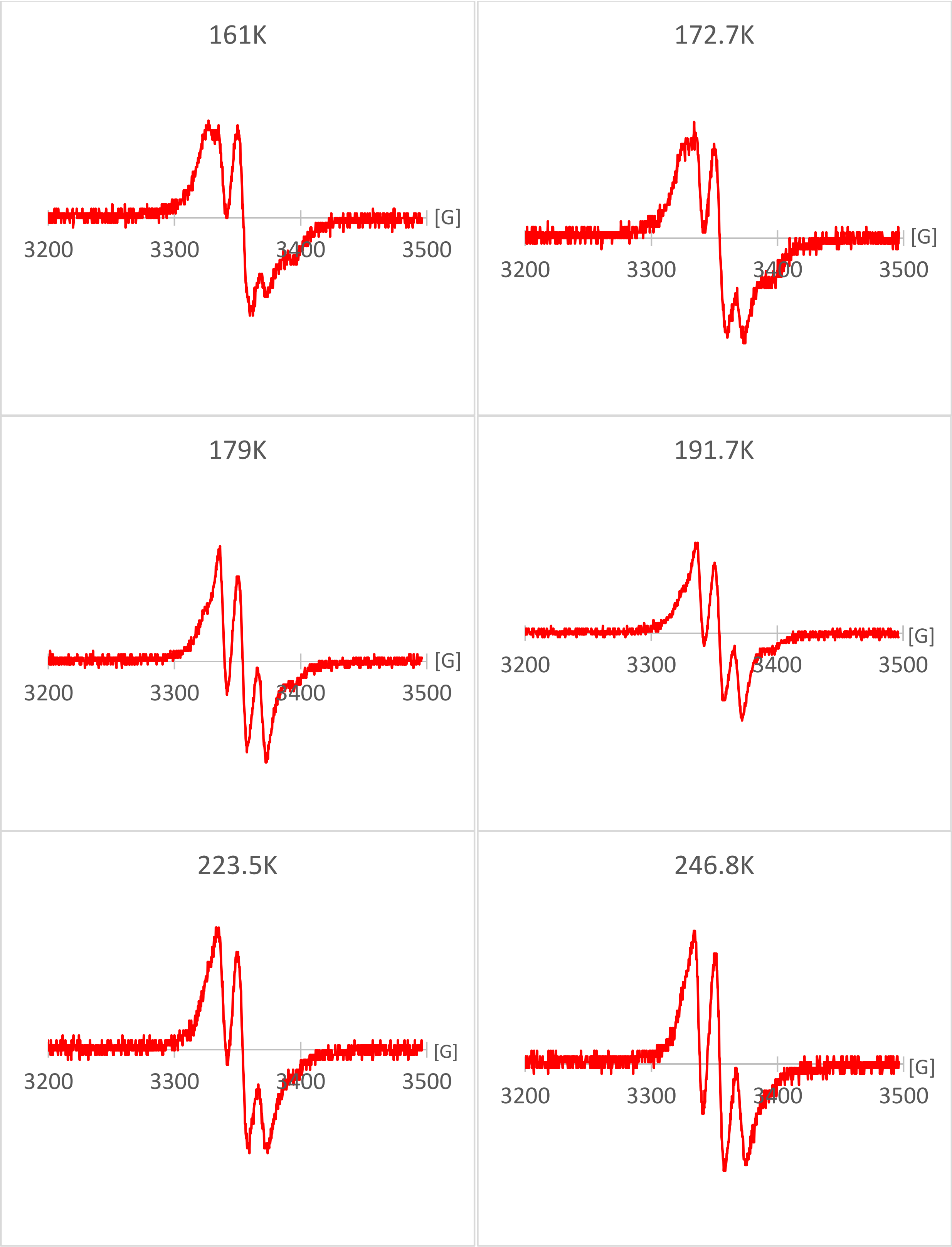
161K
172.7K
179K
191.7K
223.5K
246.8K
3200
3300
3400
3500
[G]

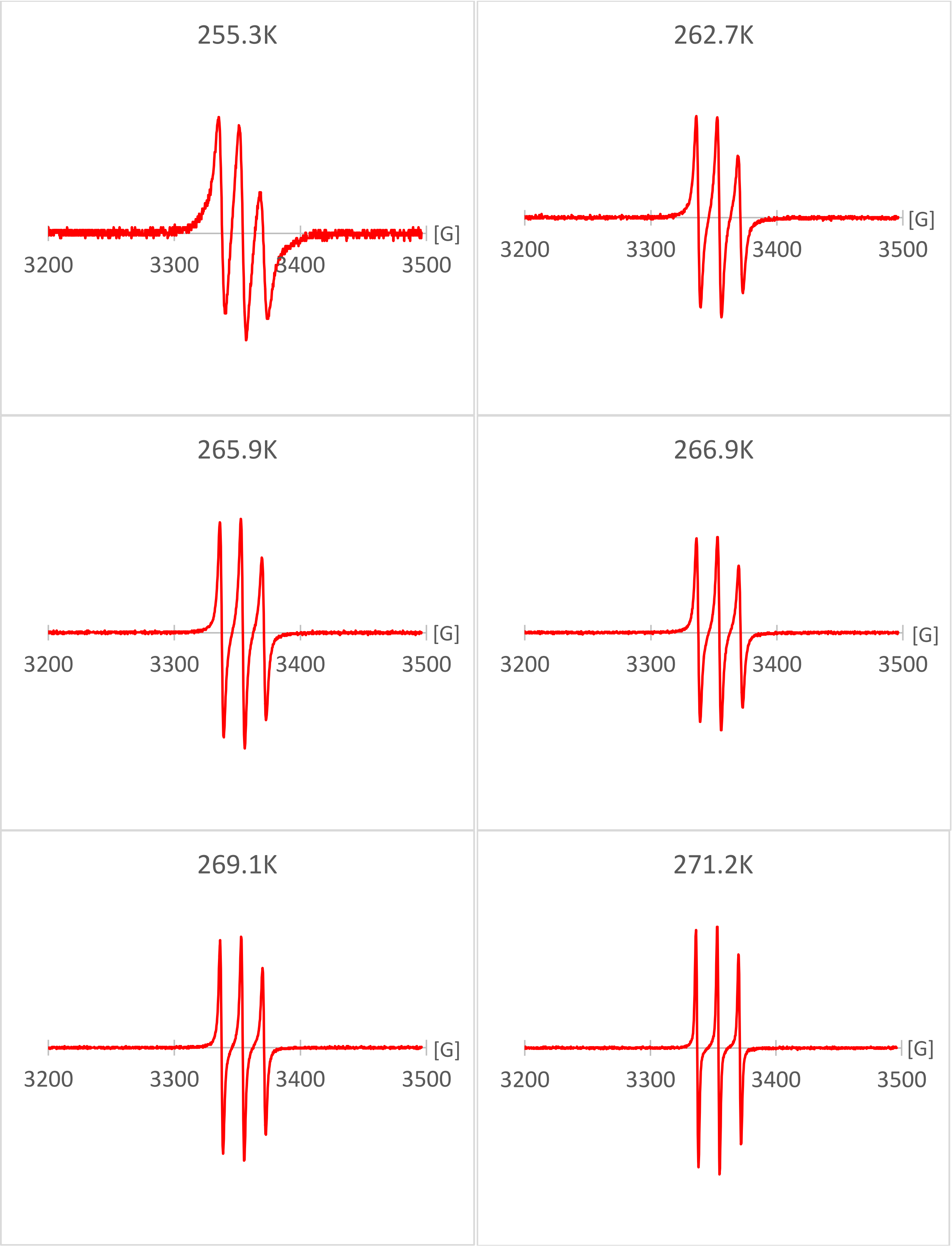
255.3K
262.7K
265.9K
266.9K
269.1K
271.2K
[G]
3200
3300
3400
3500

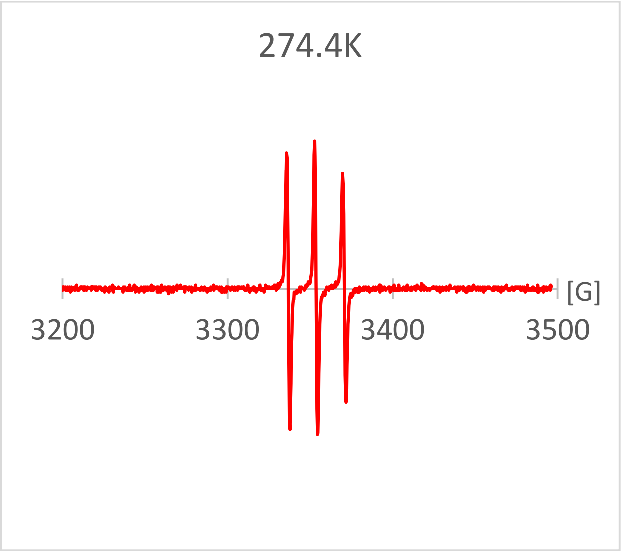

(b) Heading Process

Figure 9. VT EPR spectra of MSL spin labeled HPLC6 A20C solution at concentration of 2.0 mg/ml through the cooling process (a) and through the heating process (b).

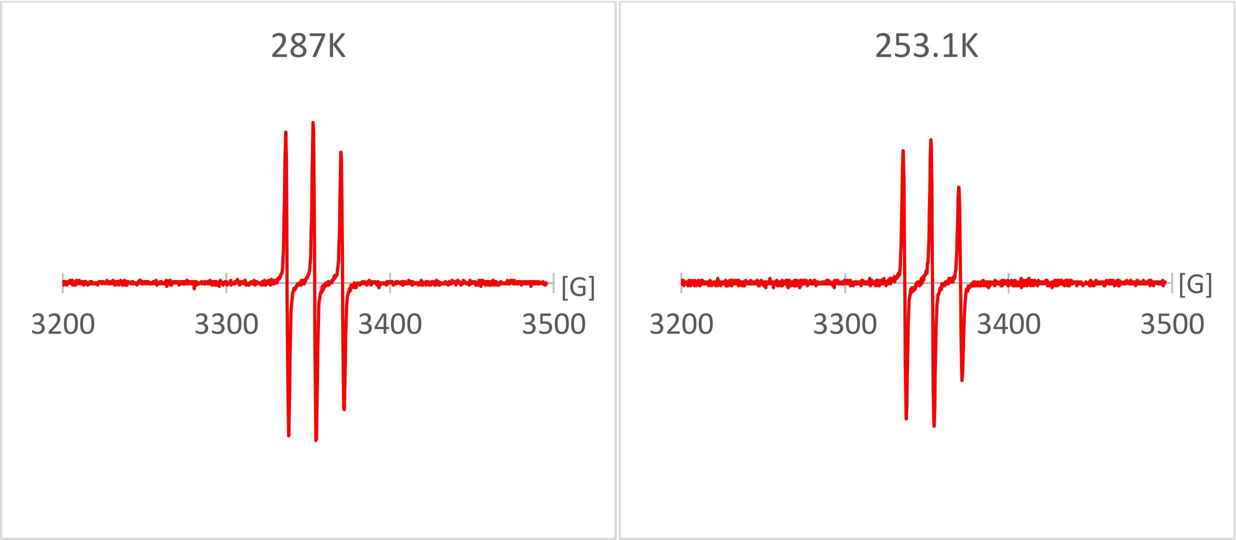

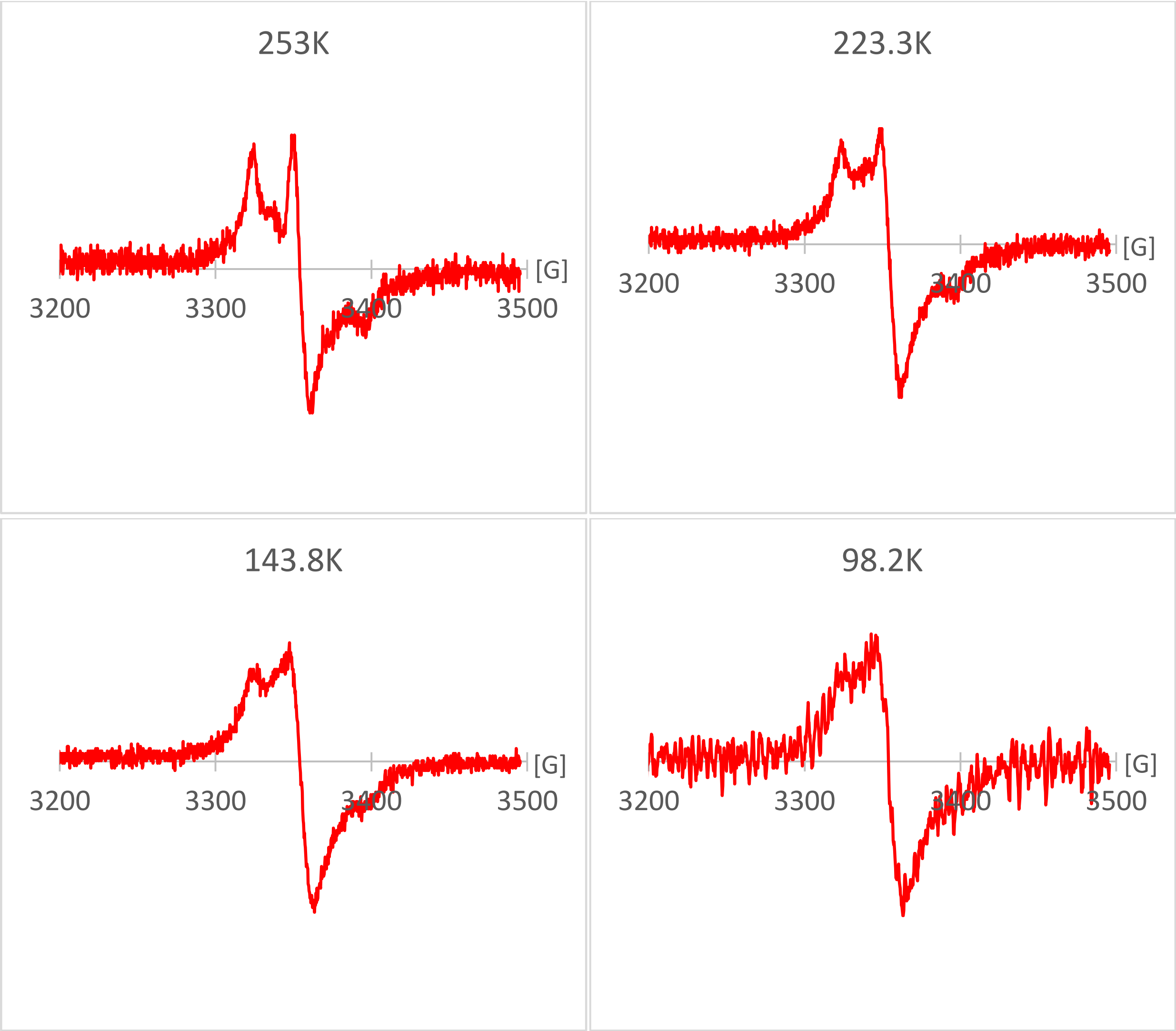

(a) Cooling Process

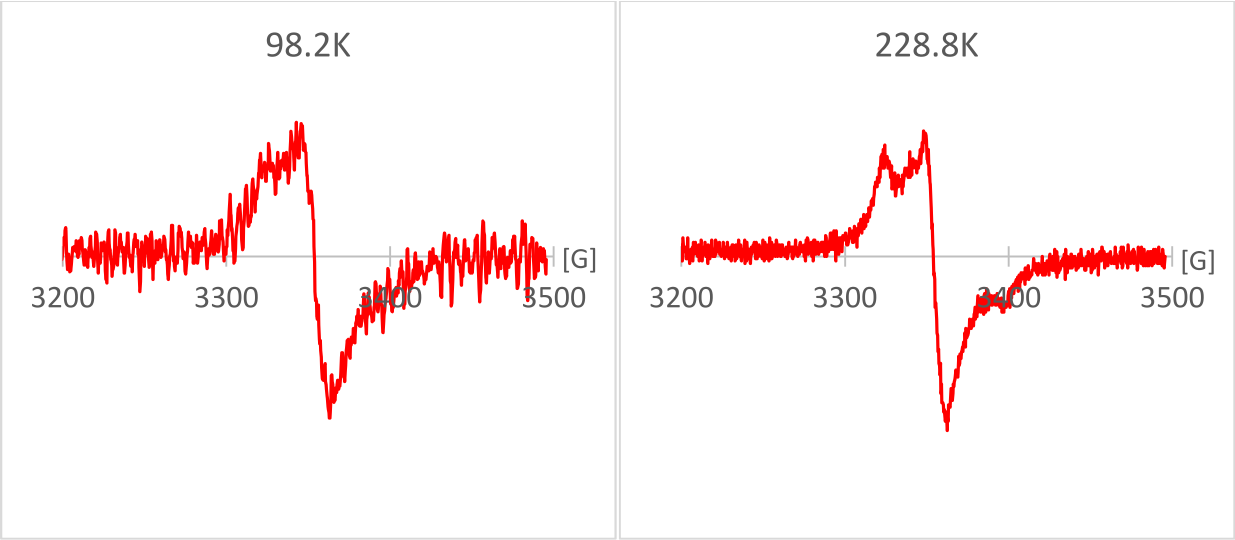

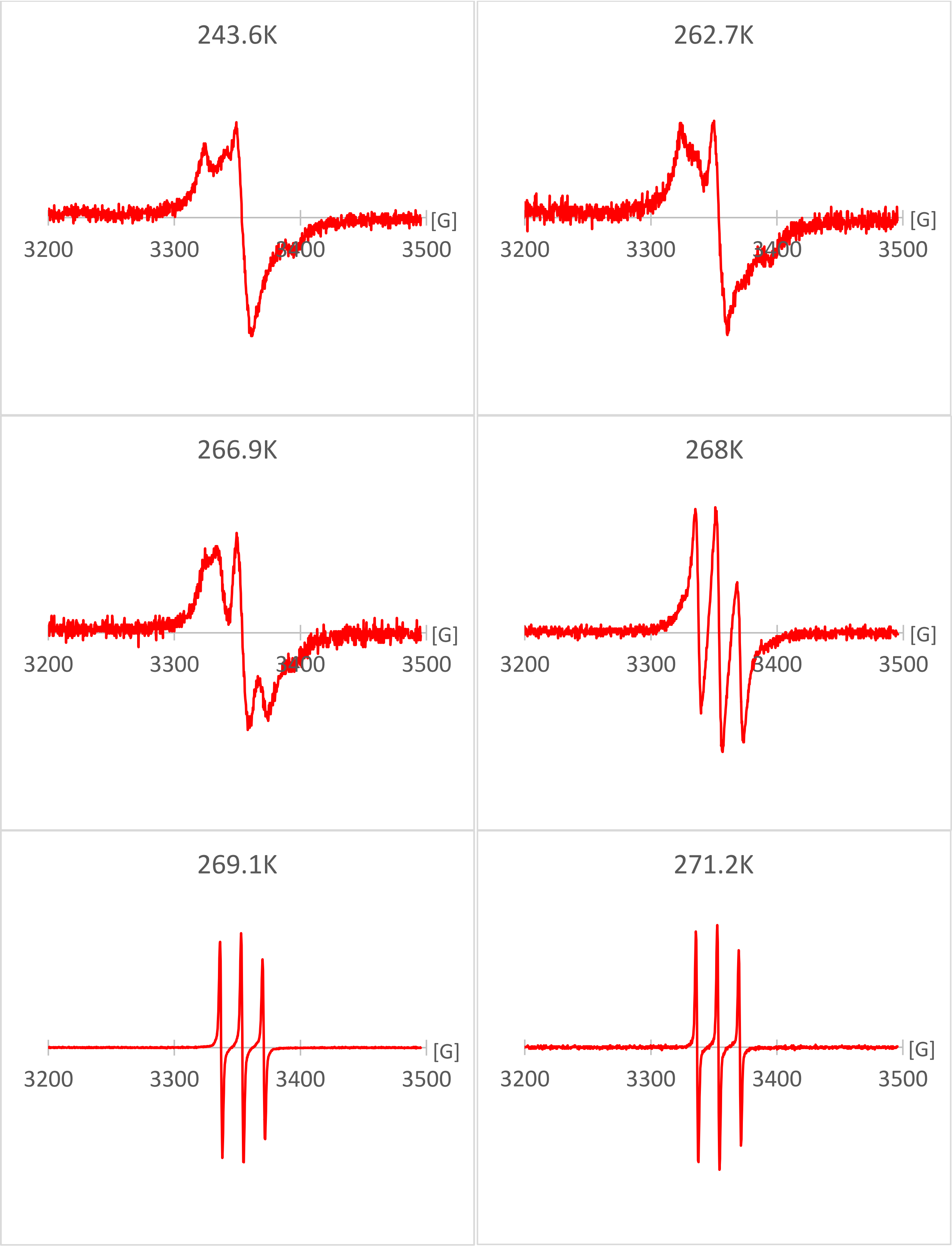
243.6K
262.7K
266.9K
268K
269.1K
271.2K
3200
3300
3400
3500
[G]

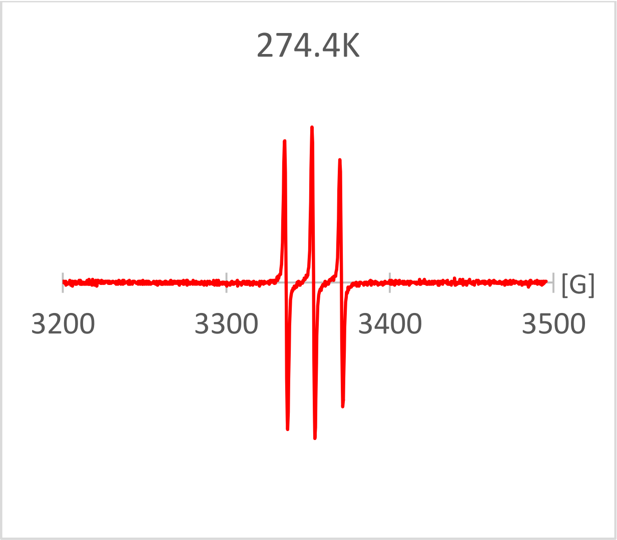

(b) Heating Process

Figure 10. VT EPR spectra of MSL spin labeled HPLC6 A11C solution at concentration of 2.0 mg/ml through the cooling process (a) and through the heating process (b).

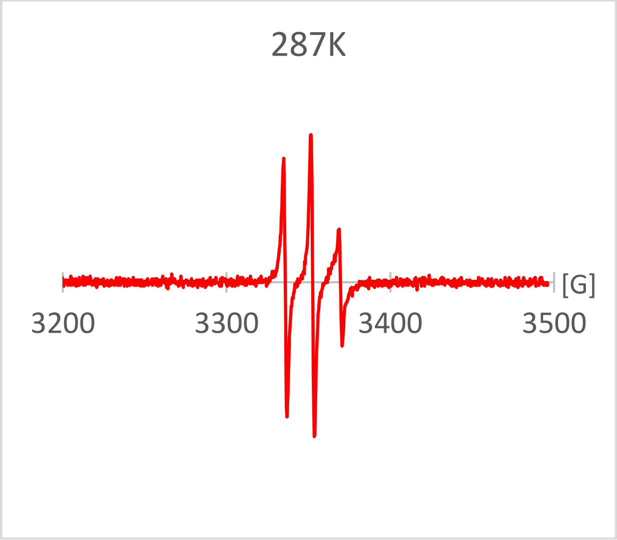

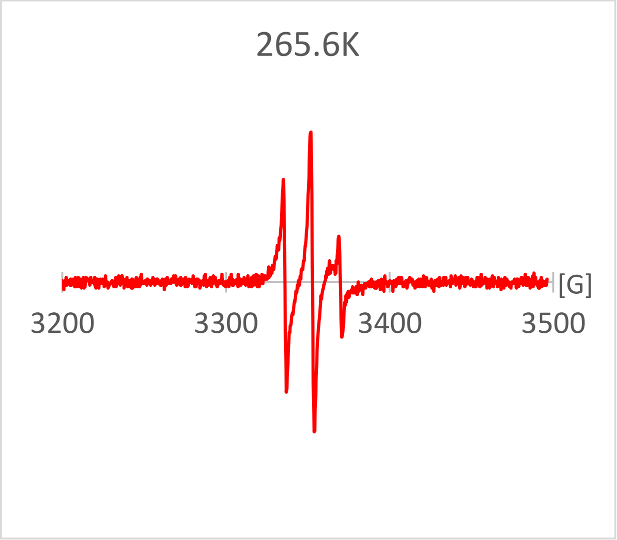

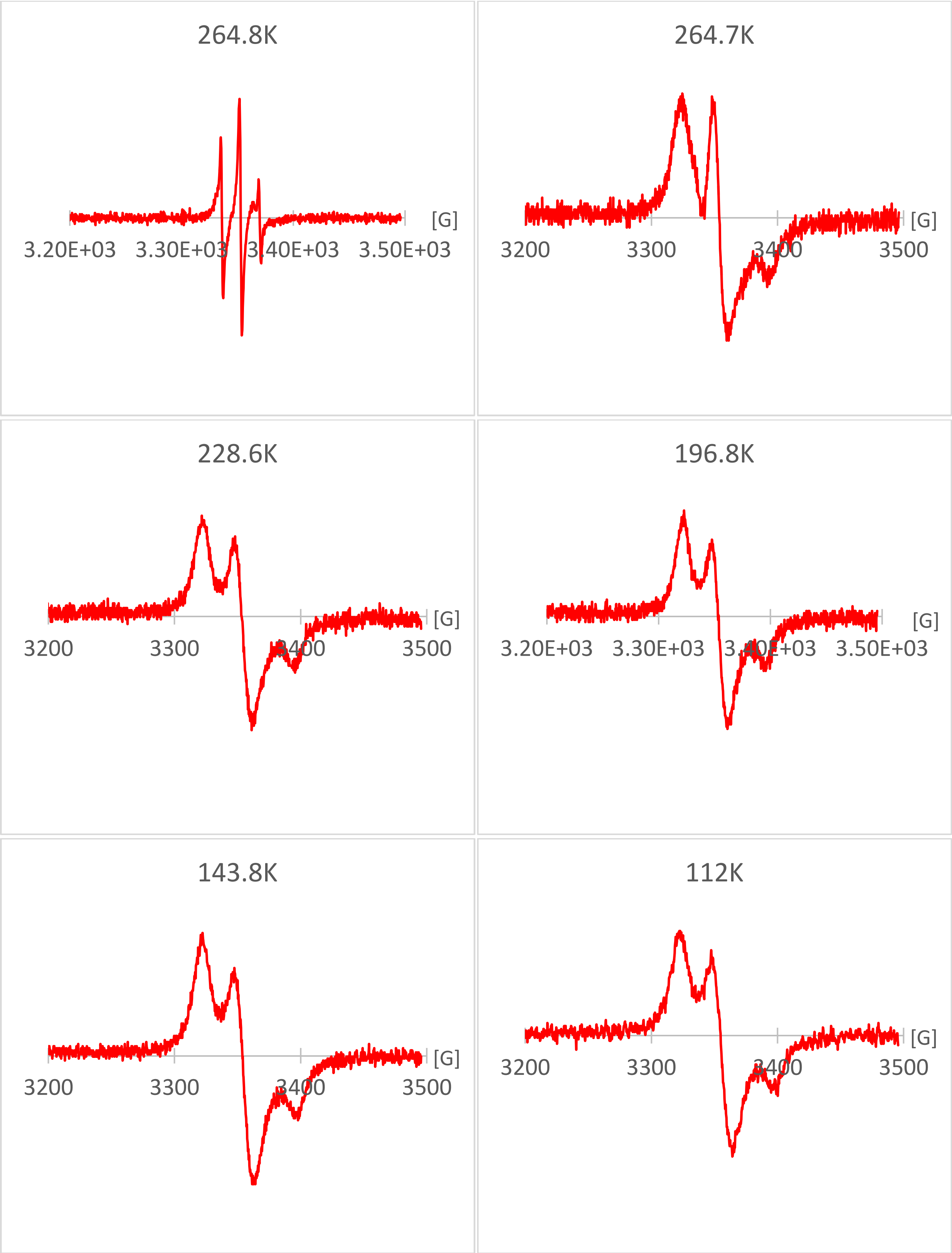
264.8K
[G]
3.20E+03
3.30E+03
3.40E+03
3.50E+03
264.7K
[G]
3200
3300
3400
3500
228.6K
[G]
3200
3300
3400
3500
196.8K
[G]
3.20E+03
3.30E+03
3.40E+03
3.50E+03
143.8K
[G]
3200
3300
3400
3500
112K
[G]
3200
3300
3400
3500

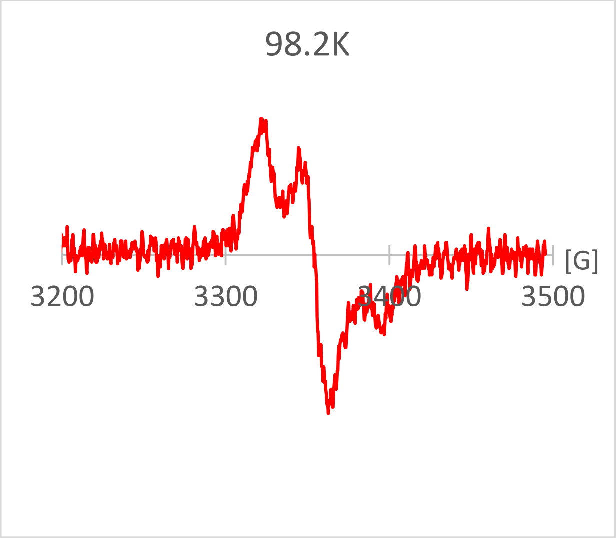

(a) Cooling Process

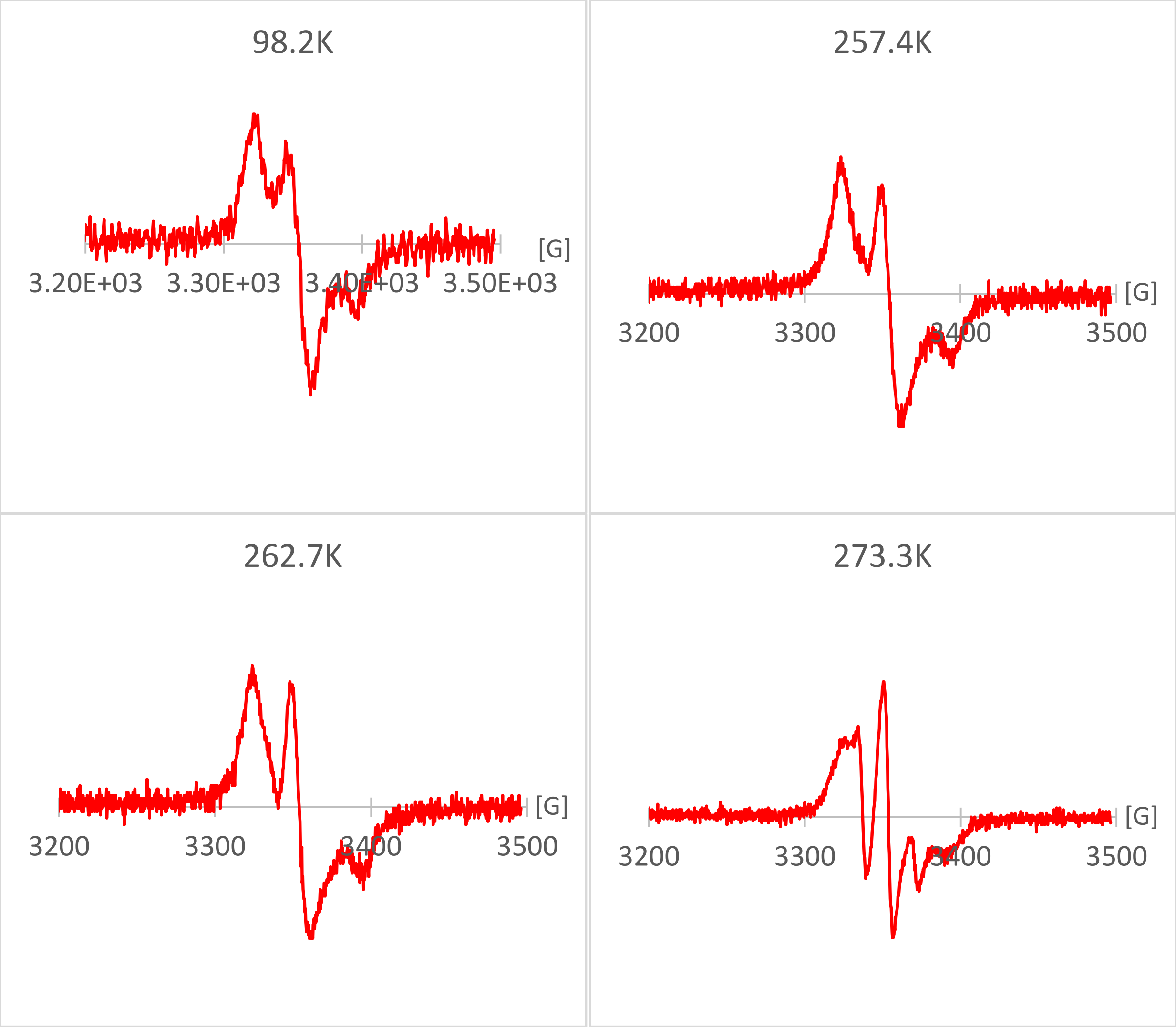

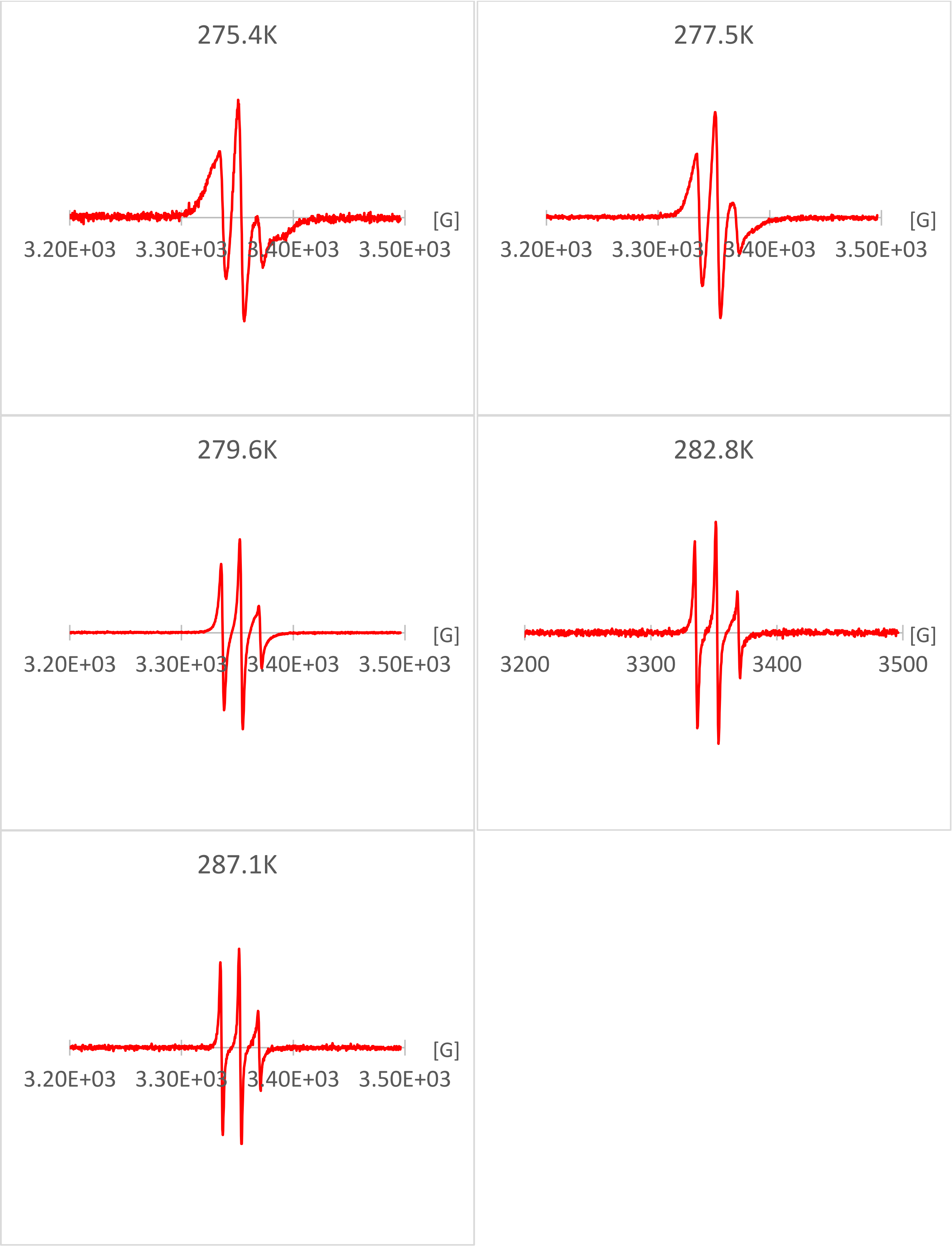

(b) Heating Process

Figure 11. VT EPR spectra of MSL spin labeled HPLC6 A17C peptide water solution at 2.0 mg/ml through the cooling process (a) and through the heating process (b).
